# Few-Shot Prediction of Toxicity of Ionic Liquids Supported by Attentive Model-Agnostic Meta-Learning

**DOI:** 10.1021/acs.chemrestox.6c00022

**Published:** 2026-05-14

**Authors:** Karol Baran, Tomasz Derron, Joachim Eichenlaub, Adam Kloskowski

**Affiliations:** † Department of Physical Chemistry, Faculty of Chemistry, 431446 Gdansk University of Technology, Narutowicza Str. 11/12, 80-233 Gdansk, Poland; ‡ Department of Dental Prosthetics, 37804 Medical University of Gdansk, Sklodowskiej-Curie St. 3c, 80-210 Gdansk, Poland

## Abstract

Prediction of chemical compounds’ toxicity enables efficient and rapid screening at the cost of utilizing experimental data as a foundation for artificial intelligence (AI) models. Given the constraints of limited data availability, few-shot learning techniques, like those based on meta-learning models, may be beneficial. In this study, the ionic liquid (IL) toxicity dataset was employed. Initially, models underwent pretraining on data from related domains before being adapted in a few-shot manner, adhering to the Model-Agnostic Meta-Learning (MAML) algorithm. The MAML algorithm utilized a neural network to predict toxicity based on the descriptors of the molecular structure. MAML enhanced the accuracy of IL toxicity prediction even with limited data, particularly when similar tasks were provided during meta-training, such as between *Escherichia coli* and *Vibrio fischeri*. Its effectiveness, however, is dependent on the diversity of the chemical space of the task for which adaptation is performed. As the final part of the study, a new version of MAML (i.e., attentive MAML) was proposed to address the limitations of the basic method. In our Att-MAML modified version of the algorithm, a part of the neural network was dedicated to adapting the importance of latent features with respect to the given test task. This approach allowed a significant reduction in standard deviation and overall improvement of the performance metrics in the low-data regime. This work advances the toxicity prediction in extremely low-data regimes beyond the current state of the art. Incorporation of attentive latent feature scaling (with Att-MAML) handles the limitations inherent to extremely low-data regimes. This novel method allows reductions in the standard deviation of metric values even while adapting on sets as small as four records.

## Graphical abstract

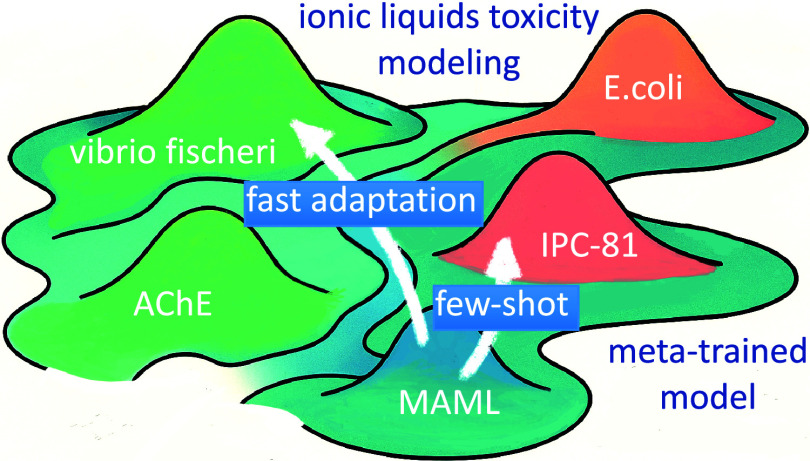

## Introduction

Toxicity modeling is a crucial component of present-day scientific research, particularly in drug discovery, chemical safety evaluation, and environmental science.^1^ Its primary objective is to assess a substance’s potential toxic effects, thereby ensuring the safety of both humans and the environment, and compliance with regulatory standards.^2^ Various standardized metrics are employed to quantify the harmful or inhibitory effects of chemical substances on biological systems.^3^ For instance, the cytotoxic concentration (CC) and inhibitory concentration (IC) represent the levels at which a compound diminishes cell viability and inhibits biological activity, respectively. The effective concentration (EC) indicates the concentration at which a specific biological effect is observed, while the lethal concentration (LC) and lethal dose (LD) denote the concentration and dose, respectively, required to cause death in a defined proportion of a test population. In antimicrobial toxicity studies, the minimum inhibitory concentration (MIC) and minimum bactericidal concentration (MBC) are utilized to determine the lowest concentrations necessary to halt microbial growth and achieve complete microbial killing, respectively.^4^

Traditionally, toxicity testing has relied on experimental approaches, including in vitro and in vivo methodologies.^5^ While these techniques are valuable, they have limitations. Comprehensive toxicity studies are costly, particularly in high-throughput screening, and they are time-consuming, which can delay the development pipeline. Additionally, ethical concerns arise from animal testing, necessitating the development of alternative methods. The rapid growth in chemical synthesis and environmental introduction makes conventional testing impractical. Conventional methodologies cannot provide timely and exhaustive safety assessments for a vast array of substances. Toxicity modeling offers a critical tool to predict toxicological outcomes efficiently and ethically, reducing the dependency on extensive experimental testing and expediting the assessment process for novel chemicals.^6^

Chemoinformatics^7^ has emerged as a crucial field in predicting chemical compound toxicity. Molecular modeling, quantitative structure–activity relationships (QSAR), and machine learning (ML) serve as instrumental approaches based on the correlation between molecular features and toxic effects.^8^ The ML algorithms, including random forests, support vector machines, and neural networks, excel in learning from extensive toxicity datasets.^9^ The utilization of machine learning algorithms necessitates the conversion of chemical structures into mathematical representations, such as descriptors, graphs, or fingerprints.^10,11^ Molecular descriptors, in particular, play a crucial role in this process by encapsulating a wide range of structural, electronic, topological, and physicochemical attributes of molecules. Notable descriptors include the partition coefficient, polar surface area, atom count, and ring system characteristics. These numerical features abstract fundamental chemical information, enabling the development of predictive models that can make informed predictions even when the complete molecular mechanism underlying the toxicity remains unknown.^12^ The algorithm training process involves refining model weights (or splits in decision trees) based on experimental data (contained in the training set). The ultimate goal is to develop a model that accurately predicts the toxicity of the unseen compounds (test set). Through chemoinformatics, researchers can conduct read-across studies that involve predicting the toxicity of unknown substances by analyzing the toxicological properties of structurally similar known compounds.^13^

The predictive capability is particularly valuable in the early stages of drug discovery and solvent development, where rapid screening and prioritization of potentially harmful compounds are crucial. Beyond academic research, chemoinformatics finds practical applications in regulatory frameworks^14^ such as REACH,^15^ the OECD QSAR Toolbox,^16^ and the EPA’s ToxCast/Tox21 ^1^ initiatives. Computational models provide a cost-effective and high-throughput alternative to conventional toxicity testing methods.^17^

Solvent toxicity modeling is of predominant importance due to its substantial environmental impact.^18^ Solvents have the potential to contaminate aquatic ecosystems and contribute to air pollution, smog formation, and greenhouse gas emissions. Furthermore, certain solvents exhibit persistence and bioaccumulation, thereby intensifying the long-term ecological damage. Researchers are currently exploring environmentally friendly alternatives, including biobased options, supercritical fluids, deep eutectic solvents, and ionic liquids (ILs).^19,20^

ILs have emerged as a fascinating group of compounds due to their “designer” nature.^21^ This characteristic allows for the manipulation of their chemical structure to achieve specific desired properties. ILs are composed of cations and anions, which facilitate the synthesis of millions of unique ionic liquid species. This vast array of possibilities makes ILs particularly intriguing for various applications.^22^ ILs are often lauded for their nonvolatility, thermal stability, and tunability, qualities that position them as potential green alternatives to conventional solvents. These properties are advantageous in numerous industrial processes where the use of traditional solvents can pose environmental and safety concerns. However, despite their low vapor pressure, ILs can still leach into water or soil during manufacturing, use, or disposal. This potential for environmental contamination complicates the assumption of their “greenness” solely based on their low vapor pressure.^23^

Certain ILs exhibit substantial toxicity to aquatic and terrestrial organisms.^24^ This raises environmental and safety concerns, necessitating a comprehensive assessment of their ecological impact.^25^ There are several structural factors that influence the toxicity of the ILs. In the case of cations, increased toxicity is associated with the presence of aromatic rings, heteroatoms in cyclic compounds, long alkyl chains, a large number of substituents, and increased hydrophobicity.^23,24,26^ Toxicity decreases for liquids based on morpholine, choline, and sulfonium cations, as well as with the presence of ester and hydroxyl substituents.^4^ Toxicity can also be reduced by the presence of hydrophilic substituents, and it is also significantly lower for liquids based on amino acids. Another group of ionic liquids considered biocompatible includes those based on choline or betaine.^27^ For anions, toxicity increases noticeably if the liquid contains fluorine atoms and long alkyl chains.^4,24^ Toxicity reduction can be achieved by using organic anions, such as carboxylates.^27,28^ Chemoinformatics modeling offers promising approaches for predicting IL toxicity across the extensive chemical space of ILs, which includes a broad spectrum of possible molecular structures. This modeling might provide insights into their potential toxicity, simplifying the development of safer and more sustainable ILs.

## Related Work

The problem of modeling the toxicity of ILs has been addressed previously in the literature. Kang et al. employed sigma profiles as molecular descriptors to model the toxicity of ILs toward the leukemia rat cell line (EC_50_ values) using multiple linear regression.^29^ Fan et al. utilized a slightly larger dataset of 155 ILs to train convolutional neural networks. Although the performance on the test set was highly satisfactory (*R* ^2^ = 0.965), their findings are somewhat constrained by the relatively small size of the dataset (approximately 40 ILs in the test set).^18^ Abdellatif et al. employed a significantly larger dataset comprising 304 ILs to construct a support vector machine model for predicting toxicity toward IPC-81.^30^ Semenyuta et al. utilized datasets on toxicity toward *Daphnia magna* (75 ILs) and *Danio rerio* (99 ILs) to obtain models predicting toxicity with *R* ^2^ metrics of approximately 0.80.^31^

The limited size of datasets necessitated the compilation of a comprehensive and extensive database on the toxicity of ILs. Yan et al. compiled a comprehensive database titled ILTox, which covers the toxicity profiles of over 1180 ILs.^32^ In addition to ILTox, Arakelyan et al. developed another database specifically focused on the cytotoxicity of ILs. This database contains data for over 1200 ILs.^33^

In a study conducted by Shan et al., data on *Escherichia coli*, AChE, and IPC-81 toxicity were utilized to develop machine learning models for toxicity prediction. The researchers achieved exceptional performance by employing the Random Forest algorithm, which operated on two-dimensional molecular descriptors.^34^ Similarly, Fan et al. employed tree-based algorithms to model the relationship between the structure of an IL and its toxicity against *Vibrio fischeri*.^35^ Wang et al. focused on neural networks and support vector machines to construct models that predict toxicity measured for IPC-81.^36^

Feng et al. conducted an analysis that demonstrated the quantity of data required for effective training of machine learning models on the ILTox database. Their findings indicated that the utilization of at least 140 ILs may lead to a plateau in the curve of the loss function plotted against the number of training instances. However, it is noteworthy that they did not explore any solutions specifically tailored for few-shot molecular property prediction.^37^

The field of low-data machine learning is gaining momentum as it is being exhaustively studied. Finn et al. proposed the Model-Agnostic Meta-Learning (MAML) algorithm that could utilize various neural network architectures to be trained on how to adapt to novel tasks even from small adaptation sets.^38^ This approach has been applied in several studies concerning chemoinformatics applications. Guo et al. utilized the MAML method operating on the Graph Neural Network (GNN) algorithm to build models predicting the toxicity of chemicals. They tested their approach in 1-shot and 5-shot scenarios, i.e., fine-tuning the classification model on one and five compounds per task, respectively. Their classification model utilized the Tox21 dataset. The MAML method allowed them to improve the quality of the models from 75.83 to 76.87 in 1-shot and from 77.18 to 78.02 in 5-shot scenarios as expressed by the Area Under the Curve (AUC) metric.^39^ Ju et al. studied Tox21 and ToxCast datasets with the MAML-GNN method. With as little as 10 samples in the adaptation set, they obtained AUC metrics of 80.21 on Tox21 and 66.79 on ToxCast datasets.^40^ Schlender et al. used a dataset comprising 24,816 aquatic toxicity values (LC_50_) for 351 different species. They utilized MAML alongside single- and multitask models.^41^ Those approaches utilized neural network models, which are often not the first choice in QSAR studies.

Currently, decision tree-based algorithms appear to be the standard for QSAR models, particularly in toxicity prediction. However, their limited compatibility with few-shot learning algorithms renders them less favorable for extremely low-data learning regimes. Neural networks frequently demonstrate suboptimal performance in molecular property prediction tasks when compared with decision tree-based methodologies. To address this challenge, several modifications have been proposed. Notably, one particularly compelling approach is independent feature mapping (IFM) proposed by Xia et al.^42^ IFM is a method designed to improve how neural networks represent molecular data. In simple terms, IFM transforms each feature independently using sine and cosine functions. This mapping allows the model to capture both low- and high-frequency variations in the data, helping it learn complex, nonsmooth relationships (like small chemical changes leading to large property shifts) more effectively.

Based on the aforementioned discussion, the questions posed in the current research are

1. Could MAML offer any added value if pretrained on the pretraining dataset containing a very limited amount of tasks?
2. Could the MAML model deliver better performance than the traditional ML approach as evaluated on the dataset regarding IL toxicity?
3. Could the MAML model be used effectively in a few-shot manner to address the issue of understudied targets?
4. Is MAML fine-tuned model performance only justified by the amount of data used to obtain and update its weights?
5. Is MAML performance only a result of adapting ILs that are known to the model for the target that the model is fine-tuned for?
6. Could the MAML algorithm be improved utilizing field knowledge on structure–toxicity relationship modeling?

## Methods

### Database and Data Preprocessing

In the present study, the ILTox database developed by Yan et al.^32^ was employed to support the investigation. The main subset of the database (subset A) encompasses data related to four principal toxicity targets, thereby providing a robust framework for toxicity assessment. Specifically, the database includes the following datasets:

- MIC for *E. coli*: This dataset comprises 125 records, detailing the MIC values that indicate the lowest concentration of a substance required to inhibit the visible growth of *E. coli*.
- EC_50_ for acetylcholinesterase (AChE): A total of 153 records are available, focusing on the EC_50_ values essential for assessing the inhibitory effects of substances on AChE, an enzyme critical for neurotransmission.
- EC_50_ for IPC-81 cells: This dataset consists of 242 records, providing insights into the cytotoxic effects of various compounds on IPC-81 cells, which are commonly used in toxicity evaluations.
- EC_50_ for *V. fischeri*: There are 165 records related to the luminescence inhibition assay of *V. fischeri*, a bioluminescent bacterium used as a sensitive indicator in ecotoxicological studies.

Toxicity assessment can be conducted on different levels of biological complexity, from the enzymes through cells and bacteria to living organisms, including fish.^25^ The susceptibility to a harmful substance identified at one level does not imply the same outcome when applied to a different one.^43,44^ Subset A includes four distinct toxicity assessment assays: one enzymatic, two unicellular bacteria, and one mammalian cell-based. Acetylcholinesterase (AChE) is the enzyme responsible for the hydrolysis of acetylocholine, which is a neurotransmitter in living organisms. Thus, AChE plays an important role in the proper functioning of the nervous system.^45^ *E. coli* and *V. fischeri* (*V. fischeri*, also known as *Aliivibrio fischeri*) are Gram-negative bacteria that are commonly used in toxicity research. The latter one is the most popular due to its natural bioluminescence that can be easily measured and interpreted. It is also one of the most cost-effective tests in the field.^4^ To assay the toxicity toward mammals, cellular lines including IPC-81, leukemia rat cell line, are often used.^46^ Additionally, cancer cell lines may be used in human oncologic research.^24^ It is worth noting that the mechanisms of toxicity toward enzymes, bacteria, and mammalian cells may vary, especially when comparing AChE with three other toxicity assessment assays.

Collectively, these datasets amount to a total of 685 records, encapsulating diverse toxicity end points across 4 distinct biological systems.

This subset of database was augmented with data collected from online ILTox resources (to form subset B of the database).^32^ These resources were utilized to determine whether the utilization of data for tasks with a low data availability would enhance the modeling process. However, the full online ILTox database contains data that required some preprocessing, unlike the previously described portion of the database. To prepare this additional part of the database for modeling, several steps were implemented. First, for each toxicological target and measure of toxicity, only one standard measure (corresponding to the toxic effect for 50% of organisms) was selected. For example, when both IC_20_ and IC_50_ values were reported for a given organism, only the IC_50_ value was retained for modeling purposes, whereas data containing solely IC_20_ values were excluded from the analysis. Second, the units were normalized to micromolar to ensure compatibility. This procedure yielded 2789 data points on toxicological targets other than *E. coli*, acetylcholinesterase, IPC-81 cells, and *V. fischeri* that were already available. These additional data points were utilized during the meta-training phase of training the MAML neural network.

This extensive data collection facilitates a multifaceted analysis of toxicological responses, thereby enhancing the reliability and comprehensiveness of the study’s findings. Instant JChem was used for data storage and management, Instant JChem v. 24.3.1, Chemaxon (https://www.chemaxon.com).

Comparison between subsets A and B is provided in Table 1.

**Table 1:** Comparison between the Full ILTox Database and Its Subset of Four Main Tasks

criteria	subset A: 4 main tasks	subset B: full ILTox DB
number of tasks	4	221
median samples per task	159	8
Min. samples per task	125	1
Max. samples per task	242	242
Std. dev. samples per task	50	26
No. of unique ILs in dataset	601	1145
organism coverage	core organisms of high relevance	broader but uneven coverage
data quality	relatively consistent and reliable	mixed quality, some uncertainty
chemical space	moderate, shared across tasks	sparse, often task-specific
meta-learning value	strong for pretraining and benchmarking	strong for few-shot adaptation and testing generalization
limitations	limited task diversity	risk of noise, overfitting, and unstable evaluation

From subset B, several low-data toxicity prediction tasks were randomly selected for additional evaluation of the few-shot learning protocol:

- *Scenedesmus vacuolatus* (EC_50_)—36 records.
- *Serratia marcescens* (MIC)—33 records.
- *Moraxella catarrhalis* (MIC)—34 records.
- *Pseudokirchneriella subcapitata* (EC_50_)—34 records.
- *Lemna minor* (EC_50_)—78 records.
- *Micrococcus luteus* (MIC)—62 records.
- *Staphylococcus epidermidis* (MBC)—42 records.
- *Proteus vulgaris* (MIC)—46 records.
- *Aeromonas hydrophila* (EC_50_)—36 records.
- *Candida albicans* (MIC)—50 records.
- *Enterococcus faecalis* (MIC)—37 records.
- *D. rerio* (LC_50_)—35 records.

### Machine Learning Modeling

In the realm of traditional machine learning modeling, the process involves the mathematical representation of molecules and the application of algorithms to discern the relationship between these representations and a target property.^7^ In this study, the target property is toxicity-assessed against a specific biological target. The molecular representations employed include the widely used RDKIT two-dimensional (2D) molecular descriptors^47^ and transformer embedding vectors derived from the pretrained ChemBERTa neural network^48^ (utilizing the Transformers Python package^49^). Transformer neural networks are renowned for their ability to compute meaningful vectors, known as embeddings, which differentiate input data.^50^ These embeddings can then be utilized by simple machine learning algorithms to estimate the target property value, a process termed the head of the classifier or regressor.

The study compared these two approaches, descriptors and transformer embeddings, with molecular fingerprints previously utilized in the literature. The algorithm selected for this investigation was the Light Gradient Boosting Machine (LightGBM), chosen for its ability to achieve higher predictive accuracy and substantially lower computational cost compared to conventional gradient boosting methods, owing to its optimized histogram-based decision tree learning and efficient parallelization mechanisms.^51^ The implementation of LightGBM was facilitated by the Python library developed by Microsoft, which provides a robust framework for this purpose.^52^

To evaluate how well the model performs, we utilized multiple standard measures for assessing its effectiveness, including the coefficient of determination (*R* ^2^—eq 1), root-mean-square error (RMSE—eq 2), mean absolute error (MAE—eq 3), and mean absolute percentage error (MAPE—eq 4), which are defined as

1 $$R^{2}=1-\frac{\sum_{i=1}^{n}{\left(y_{i}-{ŷ}_{i}\right)}^{2}}{\sum_{i=1}^{n}{\left(y_{i}-\mathrm{y̅}\right)}^{2}}$$

2 $$\mathrm{RMSE}=\sqrt{\frac{1}{n}\sum_{i=1}^{n}{\left(y_{i}-{ŷ}_{i}\right)}^{2}}$$

3 $$\mathrm{MAE}=\frac{1}{n}\sum_{i=1}^{n}\left|y_{i}-{ŷ}_{i}\right|$$

4 $$\mathrm{MAPE}=\frac{100}{n}\sum_{i=1}^{n}\left|\frac{y_{i}-{ŷ}_{i}}{y_{i}}\right|$$

where *n* is the number of observations, *y*_*i*_ denotes the actual values, *ŷ*_*i*_ denotes the predicted values, and *y̅* is the mean of the actual values.

The evaluation metrics were calculated using test sets provided in original work by Yan et al.^32^ Hyperparameters of the models were found using a validation subset that consisted of 20% of training data selected randomly.

### Meta-Learning Modeling

Meta-learning approaches, such as the Model-Agnostic Meta-Learning,^38^ offer innovative strategies for enhancing model adaptability across diverse tasks. The primary objective of MAML is to establish a robust initialization of neural network weights through meta-training on a collection of similar modeling problems (or tasks). Visual explanations of MAML principles are provided in Figure 1. This initial training phase, termed meta-training, serves as a foundation for subsequent model adaptation to a specific task of interest. The process involves collecting a variety of similar yet distinct tasks to facilitate this learning. The availability of reliable weight estimates following meta-training enables the model to adapt to an adaptation set that is significantly limited in size. This phenomenon is known as few-shot learning, where the model learns its weights from few examples. In contrast to traditional MAML, in the proposed Att-MAML model, only part of the neural network is updated in the inner loop and adaptation. Consequently, Att-MAML learns to adapt to attentive feature scaling (feature importance) rather than adapt all of the weights of the neural network that encode interactions between features.

**Figure 1:**
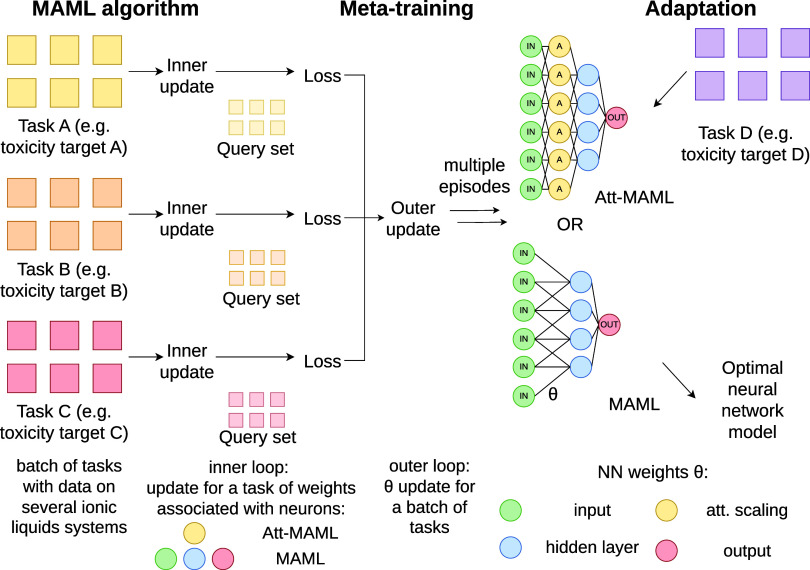
Visual explanation of the MAML algorithm.

In this study, a leave-one-task-out approach was employed as the validation protocol, as visually explained in Figure 2. This method involves utilizing three out of four tasks from the ILTox database for meta-training, while the remaining task is reserved for final model adaptation. This procedure is repeated four times, ensuring that each task is excluded during meta-training and utilized solely for adaptation. This approach simulates the scenario where researchers aim to study toxicity against organisms that have been previously understudied.

**Figure 2:**
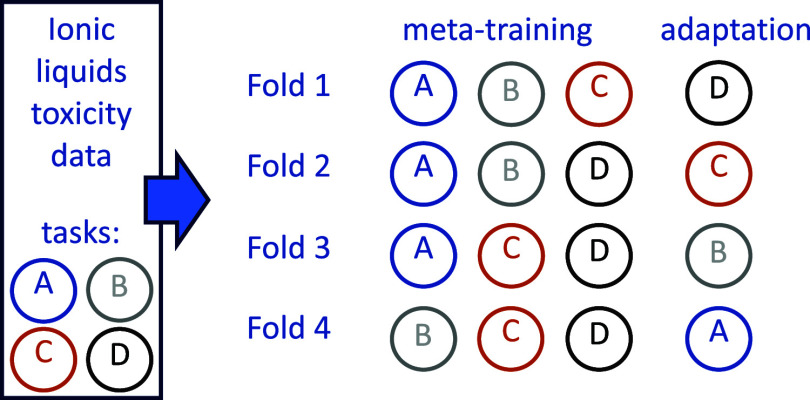
Visual explanation of the leave-one-task-out protocol.

The meta-training protocol begins with the generation of data batches, each comprising a subset of records (in this study, the record count is equal to the number of adaptation samples) from a randomly selected subset of tasks (two tasks in this study). In Model-Agnostic Meta-Learning, the optimization of the loss function is performed such that the model parameters are updated to minimize the cumulative loss across all tasks within a batch of tasks. The inclusion of a batch of tasks, rather than a single task, is essential for enabling the model to learn task-agnostic initialization parameters that can be easily adapted. For each task individually, weights are optimized through a predetermined number of gradient optimization steps (one step in this study was to increase the stability of the training process). The backward pass of the algorithm applies this gradient update across a batch of tasks, thus aiming not for the model with the highest performance but rather one that is easily trainable for the task of interest.

The adaptation step is analogous to traditional model training from scratch, with the exception that it utilizes the initial approximation of weights obtained during meta-training as a starting point. In traditional machine learning terminology, what is termed the training set is now termed the support (adaptation) set. The performance is subsequently evaluated on a test (query) set. Explanation of main MAML concepts associated with their values used in this study is provided in Table 2.

**Table 2:** Explanation of Main MAML Concepts Associated with Their Values Used in This Study

term	description	role in toxicity modeling	value in this study
meta-learning	learning to adapt to new tasks with minimal data by optimizing parameter initializations across a task distribution	model is first pretrained on diverse toxicity prediction problems and then fine-tuned for one specific task	MAML neural network
support/query sets	datasets used in inner (support) and outer (query) loops for adaptation and evaluation	datasets for deriving structure–toxicity relationships (support) and enabling generalization across diverse entities (query)	from 2 to 128 samples (*n*-shots)
inner loop	adaptation of the model for a specific task (toxicity against particular entity)	fine-tunes model parameters for specific toxicity prediction tasks	1 gradient step
outer loop	meta-optimization phase where meta-parameters are updated based on query set performance	ensures that the model learns a robust initialization that generalizes well across toxicity tasks	on batch of 2 tasks
task distribution	distribution of tasks (toxicity prediction problems) from which the model samples	represents diverse chemical scaffolds and toxicity end points for broad generalization	from 4 to 221 tasks

The MAML approach was implemented using PyTorch,^53^ based on the Deepchem implementation of MAML,^54^ to facilitate the execution of these meta-learning strategies.

## Results

### Overview of the Dataset and Traditional Machine Learning Modeling

To test few-shot toxicity prediction, the ILTox database was utilized as a source of experimental data for training and testing the models.^32^ The ILTox DB contains several tasks, and some of them (contained in subset A) have been already utilized to build independent models per task.^37^ Table 1 compares the subset of the database constructed from four main tasks (subset A) with that of the full preprocessed database (subset B). It is evident that the subset covers fewer toxicological targets (4 vs 221) and fewer ionic liquids (601 vs 1145). Conversely, the subset A of the database appears to cover core organisms of high relevance to the community, with the chemical space being shared to some extent across tasks. It is noteworthy that the number of unique ILs is not correlated with the number of data points, as for some ILs, toxicity values against multiple entities were available. In subset B, purely studied systems dominate over those well studied (a median task contains 8 samples, and the standard deviation of the number of samples per task is only 26). Consequently, the chemical space covered by tasks is often sparse, very task-specific, covering moieties and scaffolds found in the literature to be toxic or nontoxic against specific particular organisms. While the full database may not contain sufficient samples per task to allow proper generalization from the data, this dataset has the potential for evaluation of few-shot adaptation. Its applicability to meta-training warrants further testing and discussion, as limited task diversity in the subset B of the database might be a limiting factor for MAML modeling.

In addition to the statistical analysis of the tasks within the database, the distribution of the target variable warrants further examination. Figure 3 presents histograms illustrating the distribution of the target variable. The target variable varies depending on the specific task (e.g., IC_50_, EC_50_, etc.) and is consistently represented as the logarithm of the concentration expressed in micromolar units. Notably, the range of values for subset B of the database is substantially broader, as it encompasses a greater extent of the chemical space and a wider array of toxicity-related tasks. This observation may have implications for the performance of MAML models on typical tasks, such as those represented within subset A of the database, which infrequently exhibit extreme values of the target variable.

**Figure 3:**
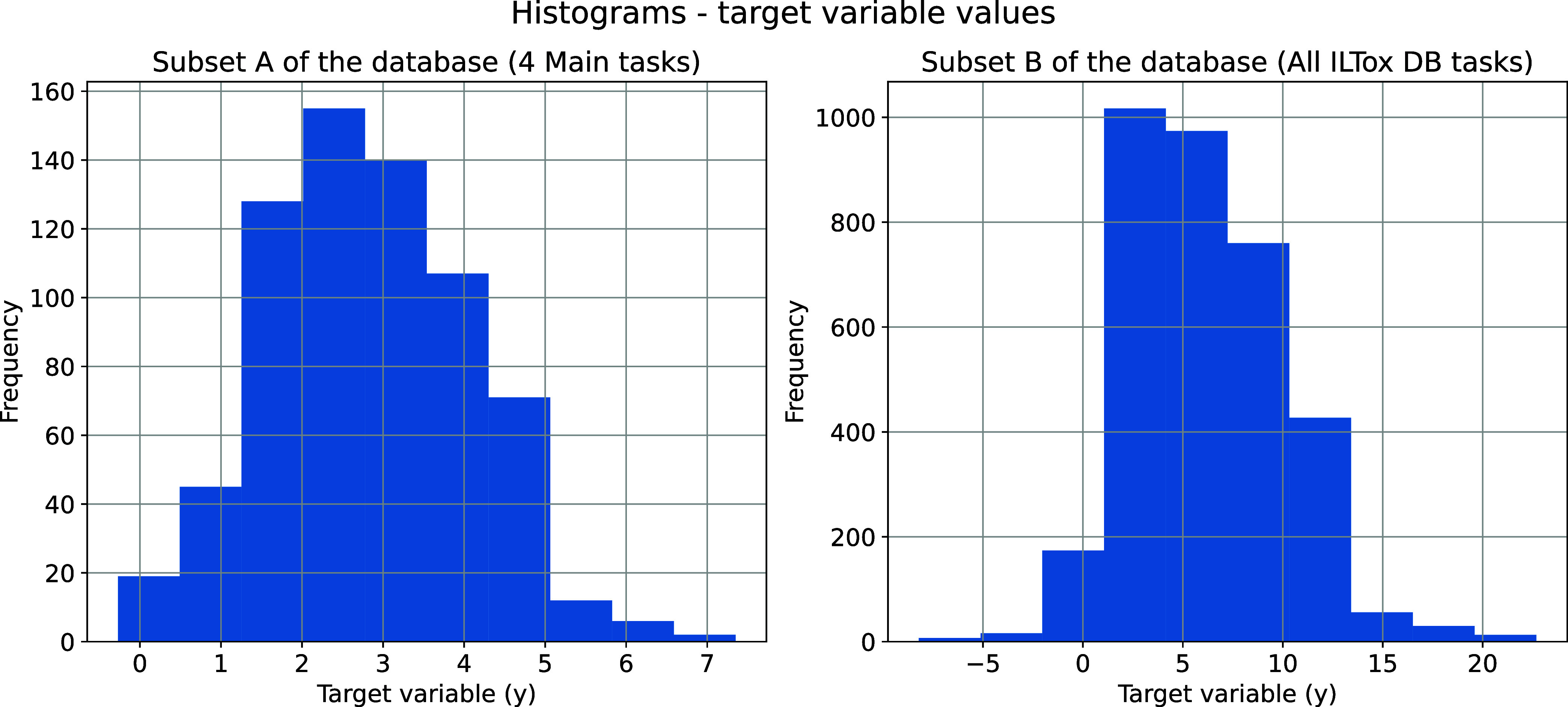
Histograms—target variable values.

Subset A is composed of four main distinct tasks. The tasks are (as explained in the Methods section) *E. coli* (125 records in the database), AChE (153 records), IPC-81 (242 records), and *V. fischeri* (165 records).

Table 3 presents the average molecular similarity of substances contained within each task in subset A of the database. To elucidate the overall similarity patterns, the coefficient was computed for all conceivable compound pairs under two distinct scenarios: within-task and between-task. The within-task scenario pertains to the arithmetic mean of similarity values derived from all pairs of compounds associated with the same task, thereby providing an assessment of intratask chemical coherence. Conversely, the between-task scenario is defined as the arithmetic mean of similarity across pairs of compounds belonging to different tasks, thereby capturing intertask structural relatedness. The methodology employed adhered to the principles outlined in our previous research.^55^ In brief, the Tanimoto similarity was computed for every possible pair of data points belonging either to the same task (within-task) or to different tasks (between-task), and the resulting values were then averaged over all combinations. It can be seen that generally, the within-task similarity was greater than that of between-task. The AChE task portion of the dataset exhibited a slightly higher within-task similarity than other tasks. Regarding intertask similarity, it seems that there were more analogous moieties and scaffolds between the AChE and IPC-81 tasks. The chemical spaces seem to be almost orthogonal to one another, however. In summary, the dataset encompasses a diverse range of compounds spanning a vast chemical space.

**Table 3:**
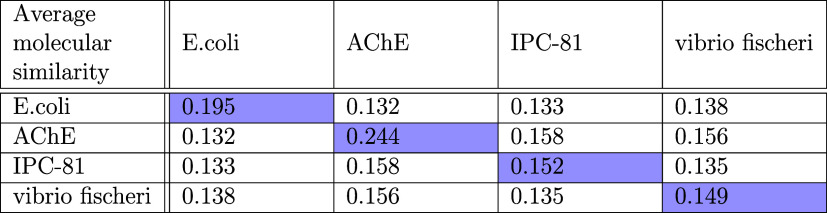
Average Molecular Similarity of the Molecules in Test Tasks (Within-Task Similarity Depicted in Blue; Between-Task Similarity Depicted in White)

All of the mentioned tasks could benefit from techniques aiming at few-shot learning due to their limited set size. However, they were previously successfully modeled, separately, each response at a time, using a classical ML approach. As a result, study on few-shot learning utilizing this dataset should begin with the evaluation of traditional learning protocols.

Table 4 presents a comparative analysis of models’ performance metrics across various molecular representations. Notably, in tasks such as *E. coli* and IPC-81, both 2D molecular descriptors and Chem-BERTa embeddings demonstrated superior performance compared to molecular fingerprints. Conversely, for tasks such as AChE and *V. fischeri*, the aforementioned representations exhibited inferior performance in comparison to fingerprints. This observation underscores the absence of a universally optimal representation that guarantees an optimal performance across all scenarios. The standard deviations of metrics are reported based on the cross-validation protocol. For the models utilizing molecular fingerprints, the standard deviations are not provided as they were not reported by Yan et al.^32^

**Table 4:** Comparison in Performance between Traditionally Trained Generalized Born (GB) Models on Test Tasks

test task	*R* ^2^ on the test task using the GB model based on 2D RDKIT descriptors	*R* ^2^ on the test task using the GB model based on transformer ChemBERTa embeddings	*R* ^2^ on the test task using the GB model based on molecular fingerprints (best scenario from^32^)
*E. coli*	0.890 ± 0.010	0.880 ± 0.021	0.80
AChE	0.801 ± 0.029	0.809 ± 0.042	0.83
IPC-81	0.808 ± 0.020	0.830 ± 0.015	0.77
*V. fischeri*	0.840 ± 0.024	0.774 ± 0.017	0.87

The primary objective of this study is to assess the efficacy of few-shot learning. To guarantee reliable insights, the models must converge to a high-performing model upon training on a full dataset. Otherwise, there is a risk of introducing specificities regarding an inefficient molecular representation, potentially obscuring the discussion. Consequently, the selected scenario should be both effective and, to minimize complexity, universally applicable across all tasks. The latter is particularly crucial for evaluating the performance of MAML models when adapted for tasks not included in the original four main tasks (in subset A, i.e., *E. coli*, AChE, IPC-81, and *V. fischeri*) but present in the ILTox database (in subset B). For these tasks, evaluation such as in Table 4 is not feasible, and the insights from the main tasks are necessary.

The preceding discussion indicated that a molecular representation capable of sustaining a consistent performance across multiple tasks should be prioritized. Consequently, molecular descriptors were selected for subsequent modeling endeavors. The two-dimensional molecular descriptors enabled the achieving of *R* ^2^ values exceeding 0.8 across all tasks, whereas neither transformer embeddings nor molecular fingerprints achieved this outcome.

### Modeling Using MAML

As mentioned in the previous sections, neural networks often underperform in molecular property prediction tasks in comparison with decision tree-based methods. To mitigate this issue, several corrections have been proposed, among which one of the most interesting seems to be the IFM method.^42^ Table 5 shows that this method allows us to obtain better performance of neural network models.

**Table 5:** Impact of IFM on Neural Network Model Performance

test task	*R* ^2^ on the test task using the GB model	*R* ^2^ on the test task using the MAML model without IFM	*R* ^2^ on the test task using the MAML model with IFM
*E. coli*	0.890 ± 0.010	0.805 ± 0.016	0.832 ± 0.026
AChE	0.801 ± 0.029	0.586 ± 0.042	0.804 ± 0.027
IPC-81	0.808 ± 0.020	0.657 ± 0.020	0.776 ± 0.041
*V. fischeri*	0.840 ± 0.024	0.562 ± 0.045	0.776 ± 0.056

### Few-Shot Learning with MAML

Figure 4 illustrates the impact of adaptation (support) set size on the performance of MAML models in terms of metrics such as *R* ^2^ and RMSE. The blue points represent the *R* ^2^ metric, and the green points correspond to the RMSE metric. The shaded regions within the graphs indicate the standard deviation values calculated from five independent adaptation set selection trials. As anticipated, the *R* ^2^ increases, while the RMSE decreases as the size of the adaptation set increases. Notably, comparable performance was achieved for the *E. coli* toxicity prediction task when fine-tuned on 32, 64, and 128 samples. Although the model fine-tuned on 16 samples demonstrates slightly lower performance, this difference is not statistically significant when considering the standard deviation. This type of variation is likely attributed to the diverse nature of the training set, from which the adaptation set is randomly selected. If 8 or 16 samples were chosen from the training set, they may not be representative of the entire task, resulting in a higher standard deviation of predictions on the test set. This phenomenon is evident in the IPC-81 and AChE tasks, where the standard deviation significantly decreases as the adaptation set size increases. However, determining the minimal adaptation set size required to achieve a model capable of generalizing effectively to the test set is task-specific and not straightforward. For the *E. coli* task, the model trained on as few as 8 samples exhibited satisfactory prediction power, likely due to the inclusion of data on the toxicity of other microorganisms (*V. fischeri*) during the meta-training phase. Conversely, this conclusion does not hold when the model is fine-tuned for the *V. fischeri* task (i.e., with *E. coli* data included in meta-training). This observation can be attributed to the vast chemical spaces present in both tasks. As previously discussed in Table 3, on average, molecules in *E. coli* tasks are more similar to each other than in *V. fischeri* tasks.

**Figure 4:**
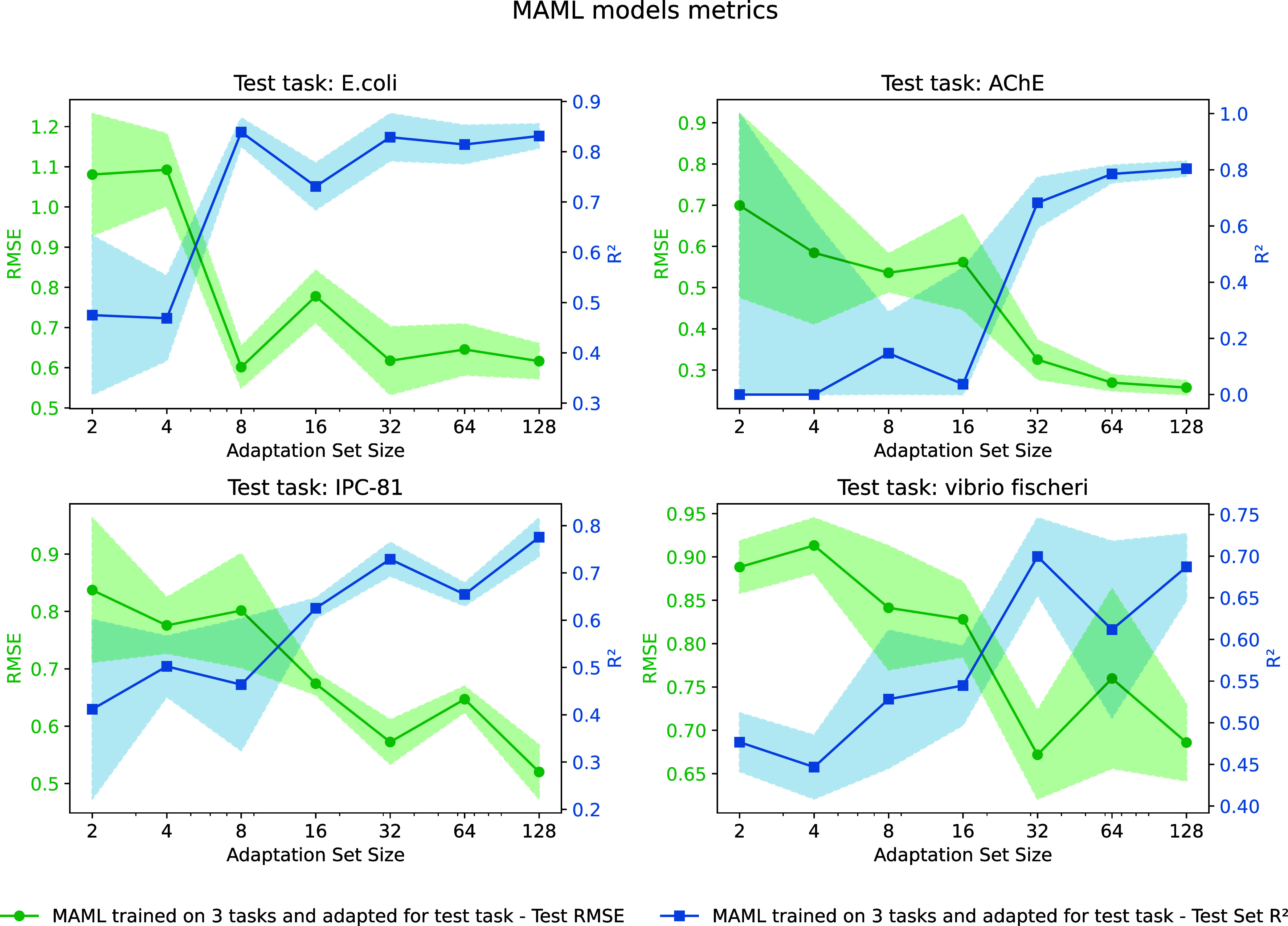
MAML model metrics.

The metrics obtained for the AChE model appear to be slightly lower in low-data regimes compared to what could be expected based on other tasks. This observation likely stems from the fact that AChE is a target biologically significantly distinct from the other tasks under investigation in leave-one-task-out validation based on four main tasks from subset A of the ILTox DB. Consequently, the MAML model was unable to extract meaningful information on how to adapt to significantly different tasks. This is well supported by the classification provided by Yan et al.,^32^ where AChE was assigned to the “Other” group when grouped by the organism family. Due to the different toxicity mechanisms of ILs toward enzymes and cells, it is expected that the results obtained for the AchE may noticeably differ. ILs may interact with enzymes and act like their inhibitors,^56^ while in the case of cells, the most probable mechanism of toxicity is a disruption of the lipid bilayer and the following cell lysis.^57^

Despite the limited size of the meta-training dataset, comprising only three tasks, additional toxicity prediction tasks can be incorporated during this phase of training. Given that subset B of the ILTox DB encompasses more than four primary tasks, alternative selection of pretraining tasks can be employed. Figure 5 illustrates a comparison of MAML model metrics when various meta-training sets were applied. It shows metrics’ evolution with respect to the adaptation set size change from 2 up to 128 data points. In the case of the *E. coli* task, the task subset of the dataset was smaller than the largest adaptation set size tested. It was decided to still use the same notation in the graphs to maintain consistency in graphs between tasks. Consequently, whenever the graph relates to 128 points in the adaptation set for the *E. coli* task, it should be interpreted as “up to 128 data points”, meaning a full training set in that instance. It is evident that models pretrained using subset B of ILTox DB generally exhibited the lowest performance metrics. This observation can be attributed to the fact that the same IL can manifest significantly varying toxic effects when it is evaluated on different targets. Furthermore, some of the tasks within subset B of the ILTox DB consist of a limited number of samples, potentially insufficient for the MAML to effectively learn adaptation strategies. However, a solution exists that utilizes only tasks that are similar (belonging to the same family of biological entities) to the test task. This scenario occasionally enhances the models’ quality, particularly in low-data regimes (e.g., when fine-tuning on 16 samples for the *V. fischeri* task). In most cases, however, the difference between this scenario and the default leave-one-task-out validation based on the four main tasks was not statistically significant.

**Figure 5:**
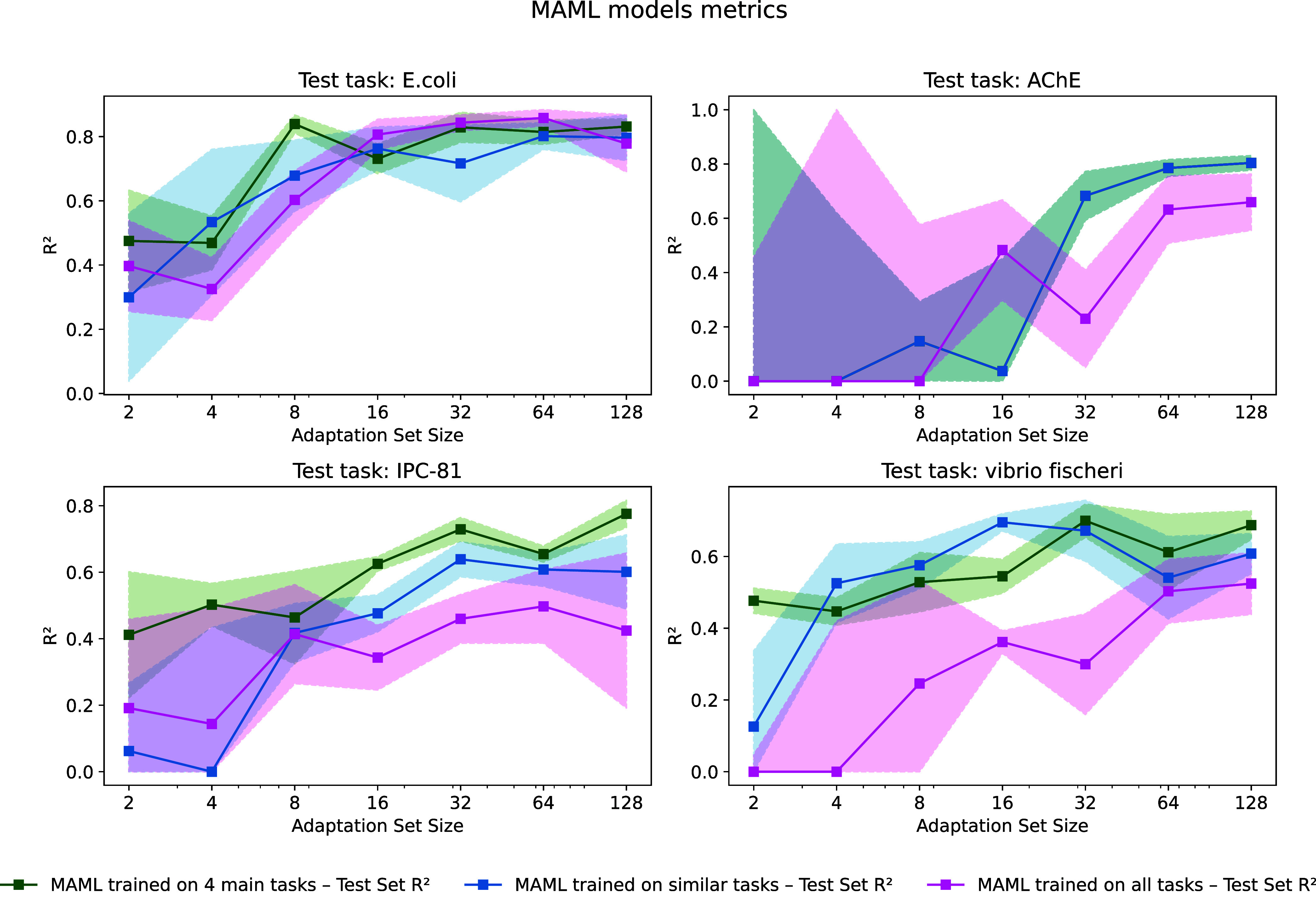
Metrics of the MAML model utilizing different meta-training datasets (green, MAML trained using only four main tasks, i.e., subset A, blue, MAML meta-trained using tasks from subset B similar to the test task, pink, MAML pretrained using all tasks from subset B).

In addition to task similarity, the utilization of data related to one common toxicological parameter should be evaluated, as there are substantial variations in toxicity expressed as EC, LC, and MIC. Consequently, the inclusion of all data (as exemplified by pretraining MAML utilizing all tasks from subset B of the database) may introduce confounding factors for the neural network model.

Figure 6 illustrates the effectiveness of MAML training utilizing this intuition. However, it does not yield a significant improvement of the *R* ^2^ metric over training using similar tasks from subset B. The difference is negligible (considering the standard deviation) for *E. coli* consistently across the full range of tested adaptation set sizes. Similarly, for the AChE task (as compared to meta-training on subset A due to the absence of similar tasks), not much added value was observed while utilizing this protocol. On the positive side, however, it was feasible to reduce the standard deviation of prediction with an adaptation set size as small as 16, enabling the model to achieve a metric value close to *R* ^2^ = 0.6. For tasks such as IPC-81 and *V. fischeri*, even a degradation of metrics compared to the inclusion of a similar task protocol was observed. Furthermore, it contributed to less stable adaptation, as evidenced by the adaptation on 64 samples in the AChE task. This observation can be attributed to the utilization of mixed-quality data with a sparse chemical space. Since the AChE dataset comprised a relatively uniform chemical space, the adaptation process was disrupted by lower quality meta-training data.

**Figure 6:**
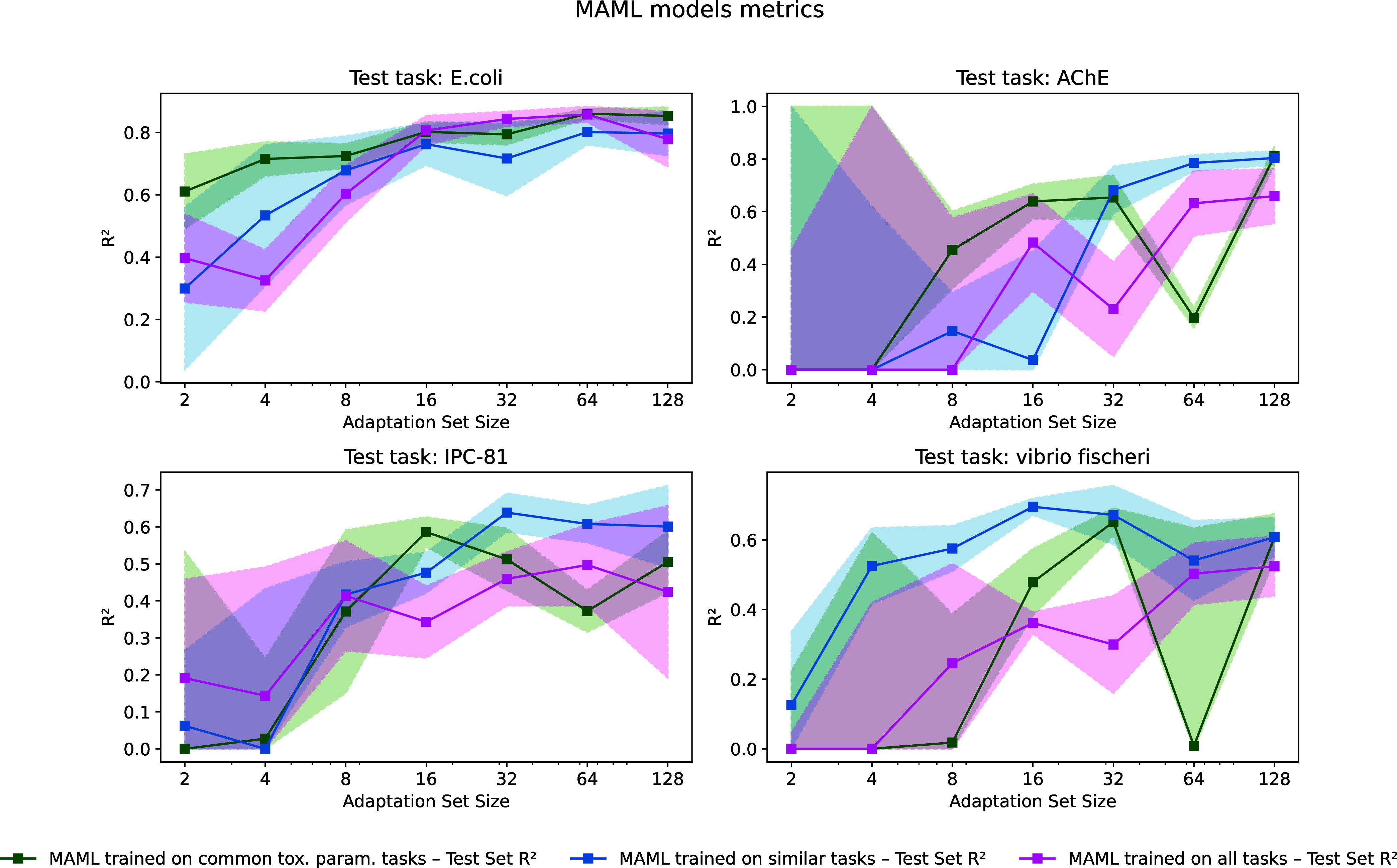
Metrics of the MAML model utilizing different meta-training datasets (green, MAML meta-trained using tasks from subset B that share the same toxicological parameter as a test task, blue, MAML meta-trained using tasks from subset B similar to the test task, pink, MAML pretrained using all tasks from subset B).

It might be hypothesized that MAML meta-trained on subset B would lead to better performance if it was meta-trained on the directions of toxicity change rather than precise value prediction. This goal could be achieved by incorporation into training elements that would more resemble classification than regression. This could be implemented by incorporating hybrid loss constituting the weighted sum of root mean squared error (RMSE, regression) and binary cross entropy (BCE, classification) losses. To make the target compatible with BCE loss, it was transformed by a hyperbolic tangent function (into the 0–1 value range) and assigned a binary label (using a threshold of 0.5 of the transformed value). The direct usage of hybrid loss for both meta-training and adaptation did not yield any gain in performance metrics, as can be seen in Appendix A. No impact of changing the ratio between RMSE and BCE in hybrid loss was observed. The alternative to utilize pure BCE during meta-training, followed by adapting with RMSE loss resulted, plausibly, in the unstable model’s training and *R* ^2^ = 0 across all test tasks and adaptation set sizes. Consequently, it was decided to study the usage of hybrid loss only during meta-training and switching into RMSE loss for adaptation.

Figure 7 shows how MAML performance changes when trained with a hybrid loss function. The two compared scenarios included protocol utilizing meta-training on a hybrid (with the RMSE-to-BCE ratio being 1:1) adapted using only RMSE loss (green plot in Figure 7) and protocol utilizing RMSE loss for both meta-training and adaptation (pink plot in Figure 7). It can be seen that utilization of hybrid loss yielded slight improvement in metrics in higher data regimes, however. For instance, for the test task IPC-81 above 16 samples or *E. coli* and *V. fischeri* above 64 samples, this approach allowed to significantly outperform the direct usage of subset B for meta-training with RMSE loss. The gain of changing the loss into a hybrid one was not of high importance, suggesting that usage of subset A for MAML modeling was preferable.

**Figure 7:**
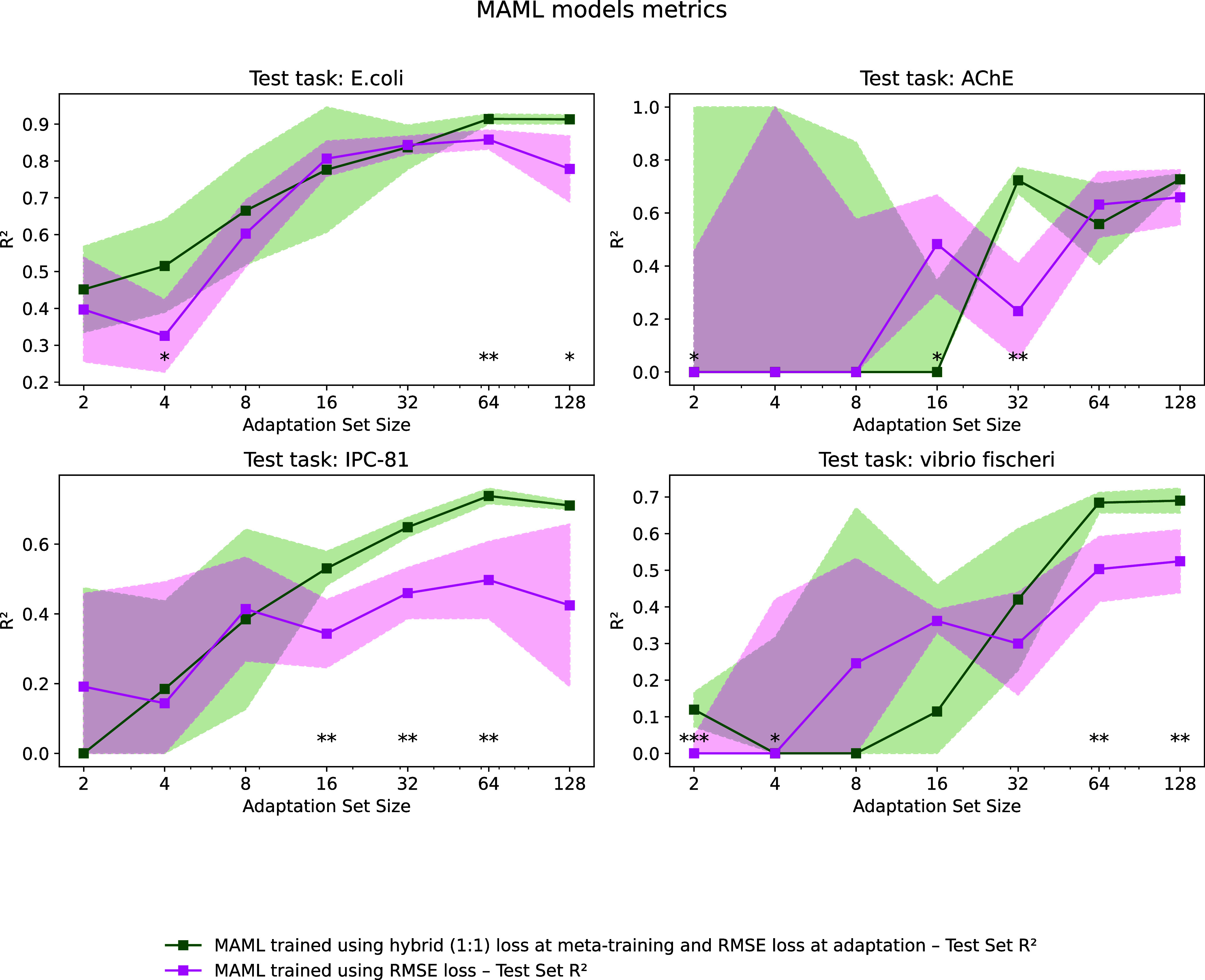
Metrics of MAML pretrained using all tasks from subset B with hybrid loss (difference significant according to the *t* test at “***” for *p* < 0.001, “**” for *p* < 0.01, and “*” for *p* < 0.05).

A comprehensive understanding of the underlying reasons for the observed modeling results necessitates a deeper analysis of their origins. While the two primary factors, namely, the relatively limited chemical space facilitating adaptation from a small number of samples and pretraining with similar tasks, have been previously discussed, the impact of common compounds between the meta-training and testing sets warrants further evaluation.

Since the test sets were independently drawn for each task, the IL contained in the test set for one task could potentially be included in the training set for another task. This inclusion could either be beneficial or misleading for the MAML model. The model utilizes a broad range of molecular descriptors to provide a foundational set. However, the values assigned to these descriptors are likely to differ substantially across various toxicity evaluation tasks.

Overall, it appears that these two effects partially cancel each other out. Figure 8 demonstrates that the inclusion of these test ILs does not significantly impact the models’ metrics. Consequently, it can be concluded that the MAML model’s strength is attributed to proper initialization based on similar machine learning prediction problems (toxicity estimation) rather than solely relying on the overlap of ILs between tasks.

**Figure 8:**
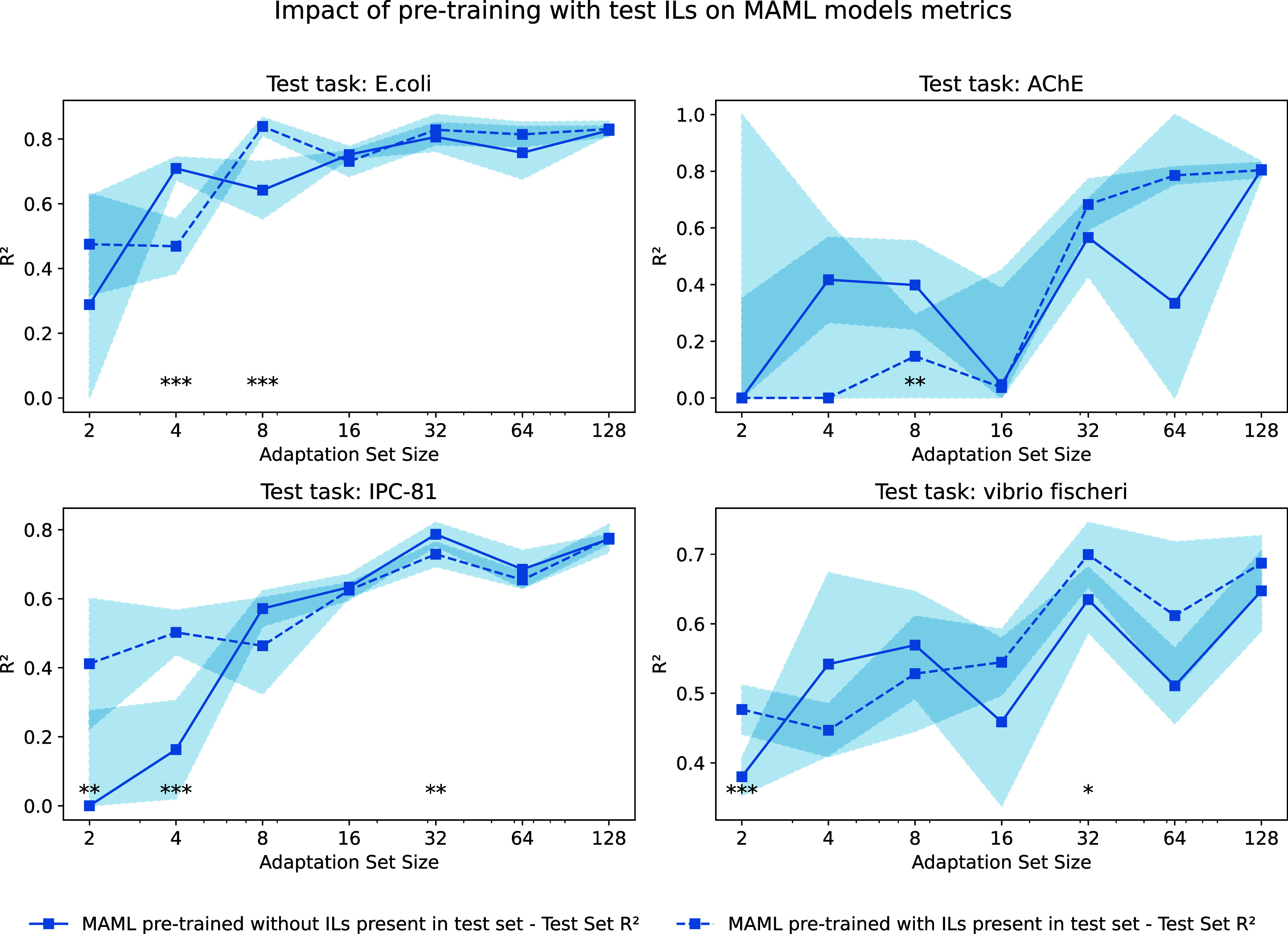
Impact of pretraining with test ILs on MAML model metrics (difference significant according to the *t* test at “***” for *p* < 0.001, “**” for *p* < 0.01, and “*” for *p* < 0.05).

### MAML Comparison with ML in Low-Data Regime

A thorough comprehension of MAML’s added value in the realm of few-shot learning necessitates a comparative analysis with conventional machine learning algorithms. While the MAML model is meta-trained on tasks other than the task used for its testing, a similar protocol might be controversial for the GB model. This is the case since the target value is task-dependent (i.e., MIC for *E. coli*, EC_50_ for AChE, EC_50_ for IPC-81, and EC_50_ for *V. fischeri*). Even if the toxicological parameter is shared, the same combination of molecular descriptors might lead to a different toxicity value as it also depends on the end point (that was not featurized in the input). Consequently, the GB model was trained utilizing only samples from the adaptation set without any additional data from different tasks to avoid confusing the model. Figure 9 illustrates the evolution of *R* ^2^ metrics in relation to varying adaptation (support) set sizes. It is evident for the GB model that the *R* ^2^ metric consistently remained close to 0 for adaptation set sizes less than 32. In scenarios with increased data availability, such as 64 or 128 samples, the GB model exhibited comparable or superior performance compared to the MAML model. Consequently, the most significant advantage of the MAML method resided in the low-data regime, where the models enabled the provision of quantitative predictions, unlike the GB model. Even with a minimal sample size of 8, the MAML model permitted qualitative predictions for IPC-81 and *V. fischeri*, as well as a quantitative model for *E. coli*.

**Figure 9:**
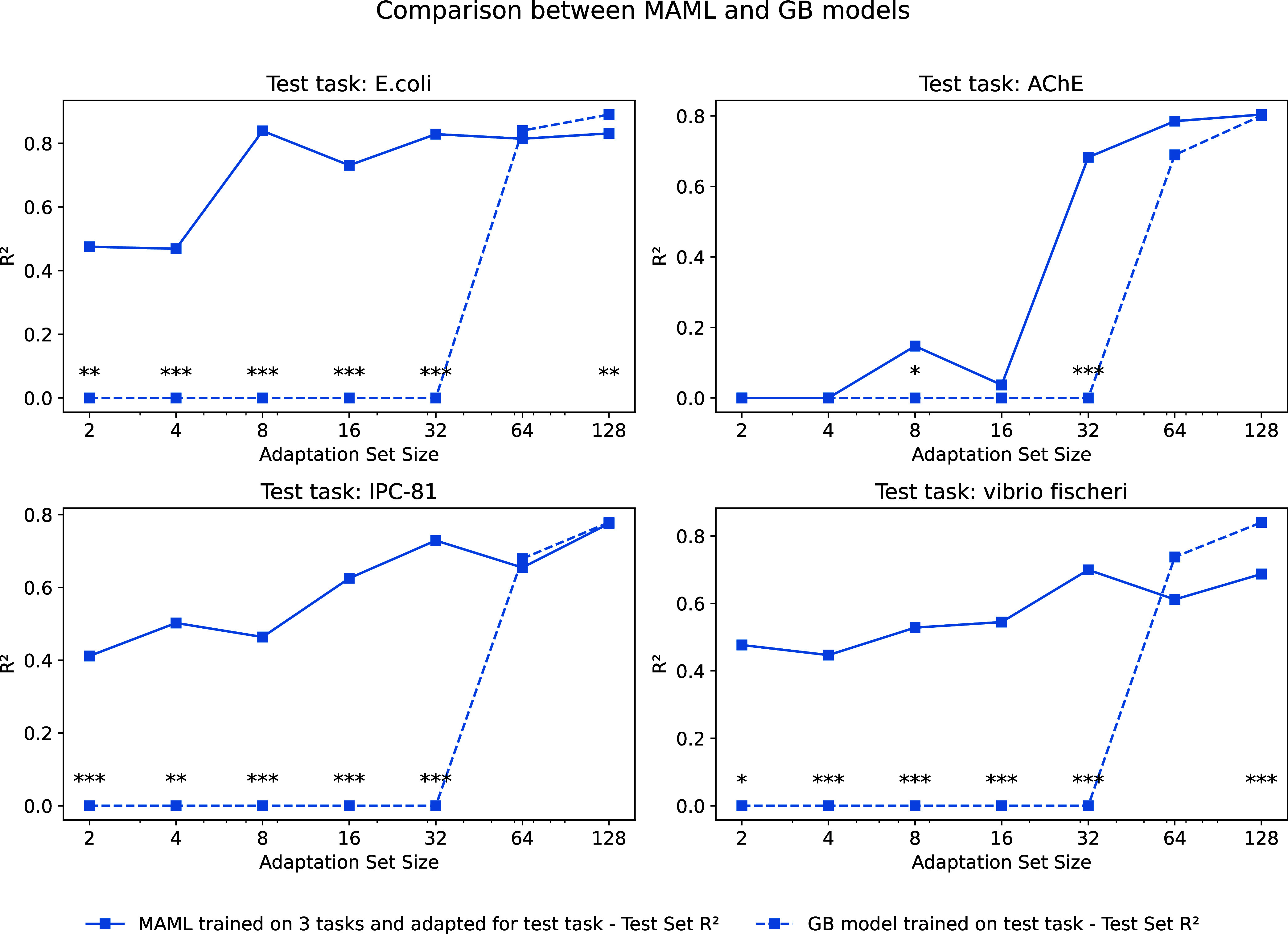
Comparison between MAML and GB models (difference significant according to the *t* test at “***” for *p* < 0.001, “**” for *p* < 0.01, and “*” for *p* < 0.05).

### Proof of Concept of MAML Toxicity Prediction from Few Data Points

The validation of a few-shot adaptation protocol for toxicity prediction from a few data points necessitates the utilization of tasks from subset B of the ILTox database, which are characterized by limited experimental toxicity values. However, for very small datasets, deviations from the normal distribution may be substantial.

Figure 10 illustrates the distribution of target variable values for 12 randomly selected 12 tasks from the ILTox database, each with a minimum of 32 samples. It is evident that this distribution significantly deviates from the normal distribution. Consequently, for this evaluation, metrics such as MAE and MAPE are preferred over RMSE and *R* ^2^.^58^

**Figure 10:**
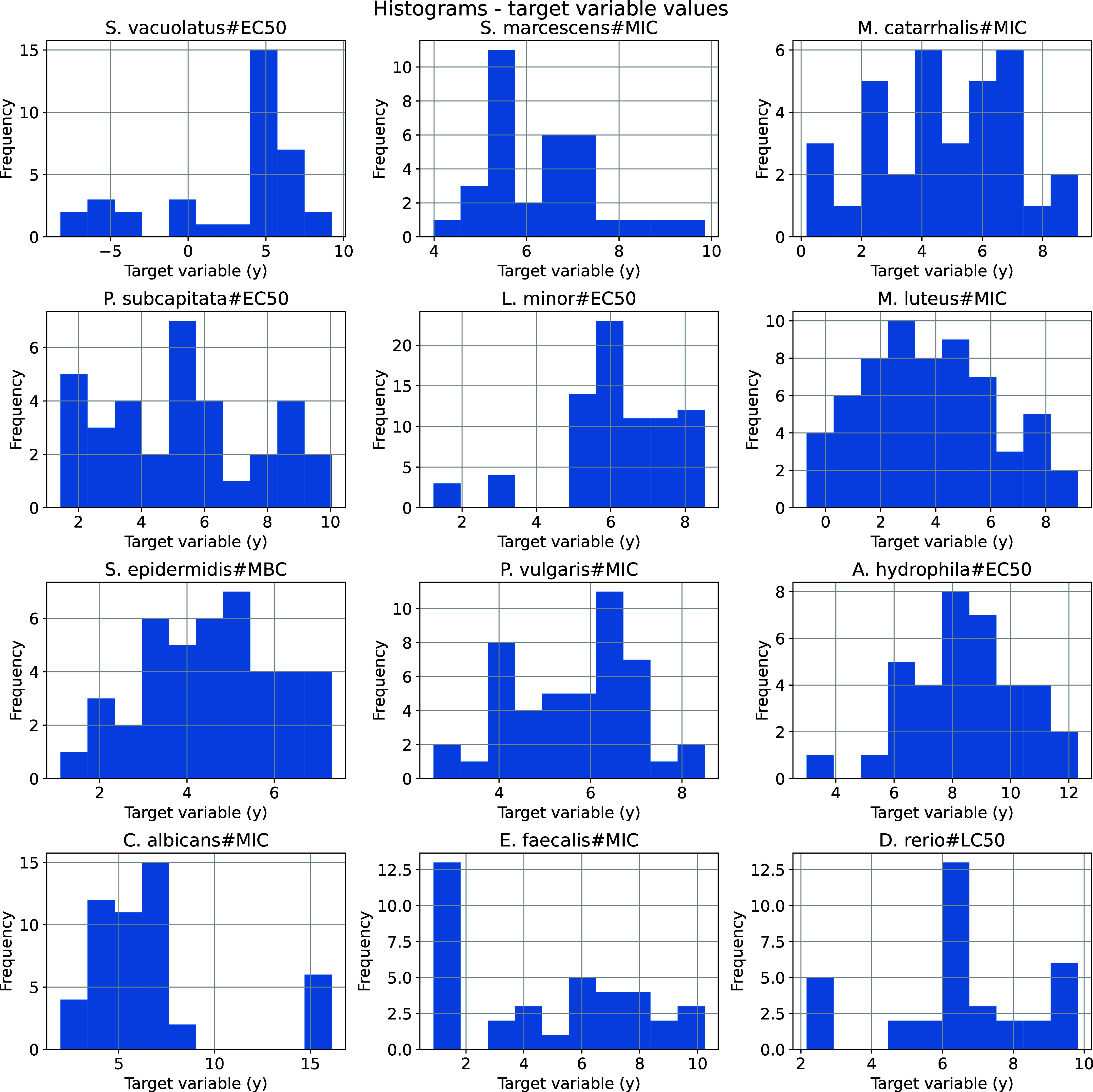
Histograms—target variable values for low-data prediction tasks.

To assess the few-shot performance of the models, query sets comprising 20% of the data for each task were employed. These query sets were utilized to evaluate models’ performance using mean absolute error (MAE) and mean absolute percentage error (MAPE) metrics. Table 6 presents the collected metrics in two adaptation scenarios: 8 and 32 adaptation samples. Notably, the MAML model, even when adapted on as few as 8 data points, frequently enables qualitative estimation of the toxicity of ILs. For 9 out of 12 tested tasks, MAPE is below 40%, indicating a certain level of confidence in the models’ estimation. As anticipated, data scarcity resulted in high variance in metric estimation. Consequently, increasing the adaptation set size from 8 to 32 samples did not necessarily lead to improved query (test) set metrics. The relatively high values of MAE suggest that few-shot prediction of toxicity remains a challenging task, yet feasible with proper machine learning techniques.

**Table 6:** MAML Model Performance on Low-Data Toxicity Prediction

test task	MAE on the query set of the test task (MAML adapted 8-shot)	MAPE on the query set of the test task (MAML adapted 8-shot)	MAE on the query set of the test task (MAML adapted 32-shot)	MAPE on the query set of the test task (MAML adapted 32-shot)
*S. vacuolatus* (EC_50_)	3.73 ± 0.87	104 ± 23	2.15 ± 0.67	51 ± 18
*S. marcescens* (MIC)	1.33 ± 0.19	21.2 ± 3.1	1.06 ± 0.20	17.7 ± 2.1
*M. catarrhalis* (MIC)	1.033 ± 0.089	39 ± 15	1.07 ± 0.35	41 ± 21
*P. subcapitata* (EC_50_)	1.90 ± 0.44	71 ± 20	0.99 ± 0.41	38 ± 29
*L. minor* (EC_50_)	1.218 ± 0.090	20.6 ± 1.6	0.99 ± 0.49	17.0 ± 8.5
*M. luteus* (MIC)	1.235 ± 0.056	30.6 ± 2.4	1.178 ± 0.043	35.9 ± 2.9
*S. epidermidis* (MBC)	1.20 ± 0.18	38.2 ± 2.1	1.436 ± 0.044	47.2 ± 3.5
*P. vulgaris* (MIC)	0.733 ± 0.043	12.8 ± 1.4	1.08 ± 0.35	17.9 ± 5.6
*A. hydrophila* (EC_50_)	2.31 ± 0.13	22.06 ± 0.98	2.52 ± 0.87	24.5 ± 8.8
*C. albicans* (MIC)	1.17 ± 0.15	25.4 ± 3.3	1.37 ± 0.12	34.0 ± 2.4
*E. faecalis* (MIC)	1.76 ± 0.38	40 ± 11	1.28 ± 0.22	35.4 ± 7.4
*D. rerio* (LC_50_)	1.44 ± 0.33	33 ± 12	1.13 ± 0.17	24.5 ± 4.2

### Improving the MAML Algorithm for Few-Shot Toxicity Prediction

The MAML protocol was shown to have several advantages over classical ML in terms of few-shot learning. It has several limitations, however. The standard deviation of the metrics was often very high for adaptation set sizes as small as 2, 4, or 8 samples. Even though rational initialization of weights was ensured by MAML, the adaptation process inseparably requires the update of more weights than there are adaptation samples (especially for 4- or 8-shot learning).

Addressing those limitations is not feasible with an unmodified protocol. For the modification to be reasonable, grounding in the chemistry of the modeled properties is advised. Since there are several moieties that have an impact on toxicity shared across multiple assays, it might be interesting to incorporate this intuition into a modified MAML protocol. MAML could be meta-trained to adapt feature importance rather than the interaction between features (molecular descriptors representing moieties). This would require the utilization of some descriptors of the assay, however. In contrast to our previous study,^59^ it is not a trivial issue to represent the toxicity end point numerically. Consequently, it was decided to rather utilize an attention-like mechanism to learn the importance of particular molecular patterns.

The chemical intuition behind this approach is that there might be moieties or their combinations that are associated with toxic effects across many assays. Their relative impact for a particular assay might differ, however. This approximation assumes that the relative importance of known structure–toxicity patterns is more important than learning task-specific patterns from few samples. Consequently, it was decided to try training the model in such a manner that the model is meta-trained on how to adapt based on varying latent feature importance.

In our modified attentive version of the MAML algorithm (Att-MAML), the neural network was split into two main parts. The first consisted of an attention mechanism that aimed at learning the relative importance of molecular features. This subnetwork was updated during the inner loop and during adaptation. The attention mechanism in this model is implemented through a small neural network that takes the latent embedding and task ID as inputs. The concatenated input is passed through an attention network, which consists of a sequence of linear layers, ReLU activation functions, and a final sigmoid activation function to ensure that attention weights have values between 0 and 1. The output of the attention network is a tensor of attention weights. These weights are then used to scale the latent embedding via element-wise multiplication, thereby modulating the feature representations in a task-specific manner. This allows the model to emphasize or de-emphasize different features depending on the specific task. This approach enables the model to adapt its internal representations to different tasks, improving its ability to be generalized across a variety of tasks. The other part of the model consisted of the rest of the neural network that was updated during the outer loop.

The Att-MAML model is visually described in Figure 1. The Att-MAML neural network contains an additional layer aiming at attentive scaling of input features. Only the weights in this layer are updated during the inner loop of meta-training and adaptation.

Figure 11 shows how metrics change with varying adaptation set sizes for traditional MAML and Att-MAML. The most important advantage of the attentive model is the reduced standard deviation of the metrics. The model provided more congruent predictions regardless of which ILs were randomly selected for adaptation. This is principally visible for an adaptation set size of 2 or 4 samples. This reduction is a consequence of fine-tuning only the weights of the attentive part of the neural network. Consequently, adaptation with a very small dataset resulted in higher utilization of meta-learned correlations and more stable adaptation.

**Figure 11:**
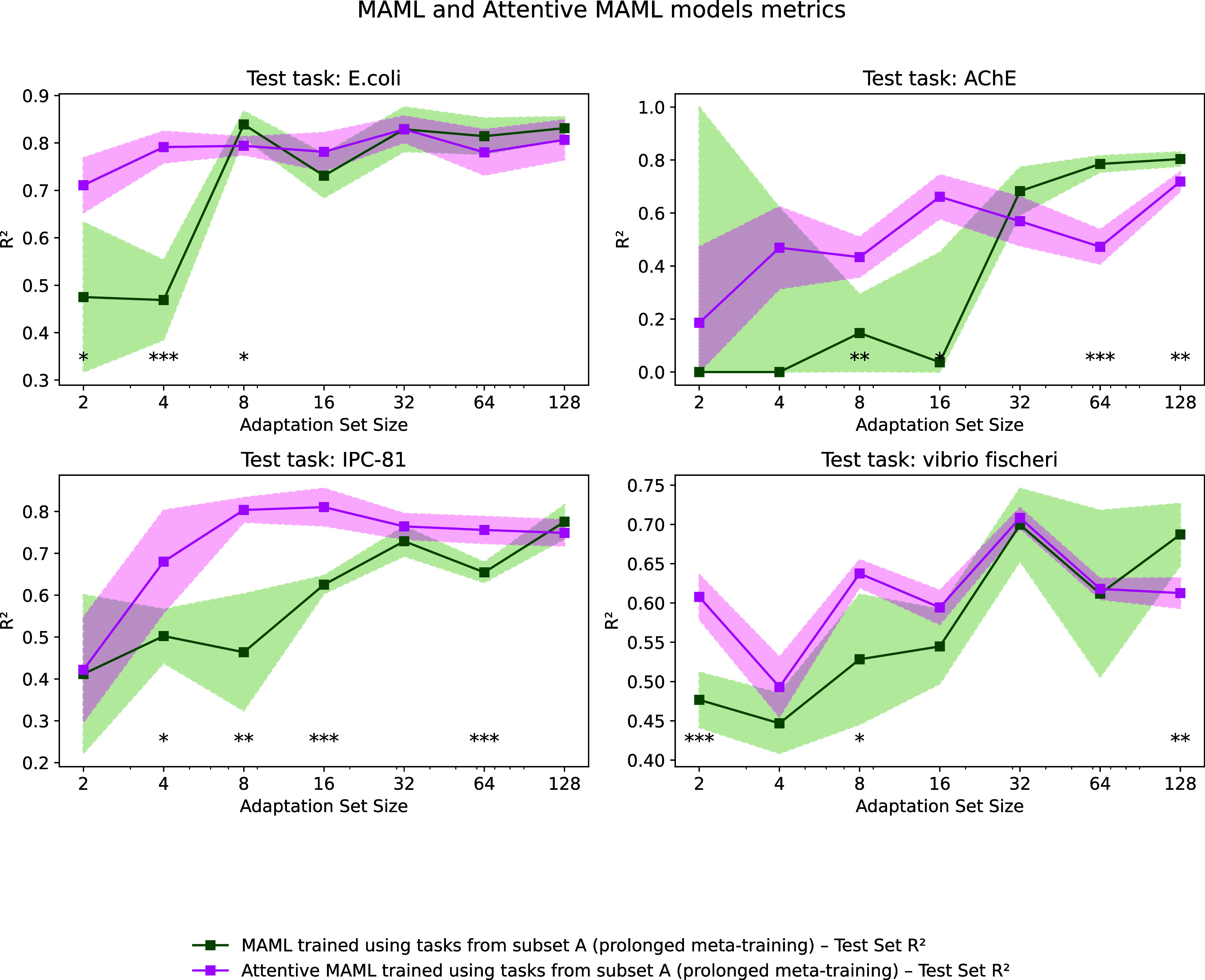
Attentive MAML comparison with traditional MAML (difference significant according to the *t* test at “***” for *p* < 0.001, “**” for *p* < 0.01, and “*” for *p* < 0.05).

The Att-MAML allowed adaptation to be more robust to randomness in the adaptation set but required prolonged meta-training for the best performance. The Att-MAML model utilizing prolonged meta-training allowed obtaining greater performance (e.g., *R* ^2^ ≈ 0.7 for the *E. coli* task at 2-shot learning and *R* ^2^ > 0.7 for the IPC-81 task in 4- and 8-shot learning). More challenging tasks, including AChE, also benefited from attentive MAML modification (e.g., *R* ^2^ ≈ 0.6 at 16-shot learning). The attentive MAML underperformed in the high-data regime for this task, however. As anticipated, Att-MAML could not learn novel structure–toxicity correlations that regular MAML could learn.

This attentive approach might require longer meta-learning. This is a consequence of changing only attentive latent features that are important in the neural network. Since the original MAML can adapt all of the weights, it could learn novel structure–toxicity relationships at adaptation. In the case of Att-MAML, it can only adjust the importance of relationships that were previously meta-learned. Appendix B shows how Att-MAML compares to the traditional method both obtained without prolonging meta-training. It can be clearly seen that the metrics’ standard deviation remained reduced compared to MAML. Prolonging meta-training was needed just to achieve better performance. However, it is important to distinguish between the impact of both the attentive approach and the prolongation of meta-training itself. Appendix C shows comparison between MAML and Att-MAML both obtained using prolonged meta-training. In that scenario, the overall performance of MAML also improved but not in a regular way. For instance, the *E. coli* model exhibited surprising degradation of metrics in the range of adaptation set sizes between 4 and 32 and enlargement of standard deviation for both very low (2) and high (128) adaptation set sizes. Consequently, prolonging the meta-training of traditional MAML did not resolve the issue of large metrics’ standard deviation but rather worsen it. The Att-MAML did show substantial improvement in the reliability and stability of few-shot adaptation.

Inherent limitations of Att-MAML should be discussed, however. As the method relies on scaling the latent feature importance, it is not expected to perform well on completely novel ions. To discuss this topic more thoroughly, a dataset on *E. coli* IL toxicity that contains ILs not present in the ILToxDB was utilized. The Att-MAML model adapted on *E. coli* ILToxDB data and tested on data from Makarov et al.^60^ A proper testing would require a testing point to be inside the applicability domain with respect to the full meta-training dataset (i.e., subset A of ILToxDB). For that purpose, the record was considered inside the domain, if the maximal Tanimoto similarity of the cation and anion to the ions in the meta-training set was larger than 0.7. A significant portion (around 58%) of these data were outside of the applicability domain as defined with the meta-training dataset, however. The remaining portion was utilized as an additional test set. The adapted model performed relatively well on it with an *R* ^2^ of 0.486 (8-shot) and of 0.519 (32-shot). However, the validation on novel cations and anions resulted in poorer performance with *R* ^2^ values of 0.00 and 0.29 on average (32-shot), respectively. The models still provided, however, qualitative predictions with an MAPE of 43.6 and 19.0% for cation- and anion-based testing, respectively. Consequently, the few-shot learning remains a challenging task for predictions on the edge of the applicability domain. It could be overcome with larger meta-training sets, as subset A of ILToxDB only contained 4 tasks and 600 records. The utilization of a larger collection is expected to strengthen the out-of-distribution capabilities of the meta-models. This analysis with respect to ILs is, however, limited by the data availability for further meta-training.

## Discussion

The research questions posed revealed intriguing insights regarding the applicability of MAML to the prediction of the toxicity of ILs. The utilization of MAML resulted in improved prediction quality even when pretrained on a limited toxicity dataset comprising a few tasks. MAML proved particularly beneficial when tasks similar to the test task were provided during the meta-training phase, such as between *E. coli* and *V. fischeri*. However, its effectiveness is somewhat constrained by the diversity of the chemical space for a specific task.

MAML significantly outperformed classical machine learning approaches in low-data regimes. The GB models failed to obtain reliable predictions (as evidenced by *R* ^2^ = 0) for adaptation set sizes below 64 samples. In this regime, MAML demonstrated a substantial advantage over GB. However, MAML did not enable the neural network algorithm to outperform GB for larger adaptation sets. MAML also proved effective for the qualitative estimation of toxicity for relatively less studied targets.

The comprehension of MAML’s advantages is a complex matter. The quantity of data alone did not justify MAML’s performance. Superior metrics were achieved when pretrained on a smaller dataset that was more closely related to the target task. More importantly, MAML’s performance is a result of rapid adaptation rather than simply operating on the chemical space provided during pretraining. Consequently, the pretraining phase serves as an initialization of neural network weights that are mathematically close to the appropriate task-specific weights rather than encoding toxicity trends that may vary between organisms.

The MAML model was shown to often provide high standard deviations of the evaluation metrics if adapted on very small sets, like 4 or 8 samples. This limitation was successfully addressed by attentive modification of MAML. By concentrating on learning latent feature importance rather than adapting all weights of the neural network, the Att-MAML model with significantly more consistent performance was obtained.

In the context of this study, several limitations in the modeling process must be acknowledged. First, the analysis was unable to provide insights into the modeling of the portion of the dataset that refers to tasks characterized by an extremely limited number of data points, such as 2 or 3. This limitation arises from the inherent difficulty in reliably evaluating models under such sparse data conditions. Consequently, the findings may not be fully applicable to scenarios where the dataset is predominantly composed of tasks with minimal data availability.

Second, the study was conducted under the assumption of utilizing small datasets with a limited number of tasks. It is essential to acknowledge that prior investigations into MAML utilization within extensive chemoinformatics applications have examined a wider array of tasks. These studies might provide more thorough and expansive insights into the efficacy of various modeling methodologies.^61^ Therefore, researchers should be mindful of these broader studies when considering the applicability of the findings from this study to their own research contexts.

While the relatively high values of metrics were obtained for limited adaptation sets (2, 4, or 8 samples per task), the model predictions should be treated with caution in an operational environment. The users that utilize the proposed method in their studies should be aware that the variability inherent to extreme low-data regimes might impact the model, especially with a limited variability of the chemical space in the adaptation set.

It should be acknowledged that the exact attribution of the performance gains to meta-learning or knowledge gaining during pretraining is impossible in this context. The joint multitask training would produce meaningless predictions, as the target variable changes from task to task. The comparison between meta-learning and classical large-scale pretraining and fine-tuning (often utilized in deep learning) requires different datasets, where joint training on multiple tasks is feasible.

The primary objective of this study was to evaluate the performance of MAML in a low-data regime. For those seeking to develop the best predictive models, it is recommended that one explore established techniques aimed at enhancing model quality. This could involve investigating the potential benefits of model ensemble approaches for few-shot toxicity learning or examining a wider array of algorithms and descriptor types. By doing so, researchers may be able to overcome some of the limitations inherent in low-data scenarios and achieve even more robust predictive models.

The future outlook is promising, particularly in the realm of ionic liquid toxicity research. Through its contribution to the current scholarly discourse, this study is anticipated to augment the assessment of the toxicity of ionic liquids. This advancement is crucial, as it will facilitate the development of more accurate and comprehensive models for predicting the potential health and environmental impacts of these compounds.

Additionally, this study has the potential to simplify the transfer of knowledge across various toxicity targets. This is important because it enables researchers to use insights from one study to guide research in other related fields. Such applications can result in more efficient and effective research processes, ultimately speeding scientific progress.

## Summary

Artificial intelligence is enhancing the efficiency and precision of predicting the toxicity of chemical compounds, especially solvents, by offering a faster and more cost-effective alternative to traditional methods. Despite its promise, accurate predictions require extensive experimental data, which is currently being addressed through techniques such as few-shot machine learning.

By employing the ionic liquids toxicity dataset in conjunction with the Model-Agnostic Meta-Learning algorithm, a comparative analysis was conducted between conventional machine learning approaches and few-shot learning techniques.

The research identified intriguing insights into the applicability of MAML to predicting IL toxicity. MAML enhanced prediction quality even with a limited toxicity dataset. It benefited from tasks similar to the test task during meta-training, such as between *E. coli* and *V. fischeri*. However, its effectiveness is contingent upon the chemical space’s diversity. Notably, MAML outperformed classical ML in low-data regimes as GB models failed to make reliable predictions for adaptation set sizes below 64 samples. Nevertheless, MAML exhibited a limitation when applied to larger adaptation sets, where GB demonstrated a superior performance. Comprehending MAML’s advantages is a complex matter, as data quantity alone did not justify its performance. Superior metrics were achieved with a smaller, more relevant dataset. MAML’s performance is attributed to rapid adaptation, not solely to pretraining.

The MAML model often resulted in obtaining metrics with very high standard deviation stemming from the impact of which compounds were randomly selected to adapt the model. This effect was especially significant for small adaptation sets composed of 2, 4, or 8 ILs. Even though the initialization of NN weights obtained via meta-training was specifically obtained to be easily adaptable from few samples, during fine-tuning, many weights had to be updated. Consequently, modification of the original method as an attentive MAML (Att-MAML) was proposed in this study. In the modified approach, during the inner loop, only part of the neural network adapting the importance of latent features was updated. This approach allowed us to obtain models more robust to the randomly selected adaptation set. The improvement in metric values and decrease of their standard deviation showed how Att-MAML could be more suitable than the original algorithm in structure–toxicity modeling.

## Supplementary Material



## Data Availability

The underlying code for this study is available and can be accessed via the link to the GitHub repository at https://doi.org/10.5281/zenodo.19278331. The files containing requirements on freely available packages needed to reproduce the work are provided in the repository. Data sources are provided in the manuscript text.

## Notes

Additional graphs on models’ comparison (PDF)
