# Supplementary material for: Few-Shot Prediction of Toxicity of Ionic Liquids Supported by Attentive Model-Agnostic Meta-Learning
Source: Chem Res Toxicol. 2026 May 14;39(6):1123–40. doi: 10.1021/acs.chemrestox.6c00022 (PMC13299051; doi:10.1021/acs.chemrestox.6c00022)

Supporting Information: Few-Shot Prediction of Toxicity of Ionic Liquids Supported by Attentive Model-Agnostic Meta-Learning

Karol Baran,[*,†] Tomasz Derron,[†] Joachim Eichenlaub,[‡] and Adam Kloskowski[†]

†*Department of Physical Chemistry, Faculty of Chemistry, Gdansk University of Technology, Narutowicza Str. 11/12, 80-233 Gdansk, Poland*

‡*Department of Dental Prosthetics, Medical University of Gdansk, Sklodowskiej-Curie St. 3c, 80-210 Gdansk, Poland*

E-mail: karol.baran@pg.edu.pl

Phone: +48 58 347 25 93

Contents

Appendix A: Metircs of MAML pre-trained using all tasks from subset B with hybrid loss with varying BCE to RMSE ratio in hybrid loss function **2**

Appendix B: Metrics of MAML and Att-MAML models (without prolonged meta-training) **3**

Appendix C: Metrics of MAML and Att-MAML models (with prolonged meta-training) **4**

Appendix A

Metircs of MAML pre-trained using all tasks from subset B with hybrid loss with varying BCE to RMSE ratio in hybrid loss function

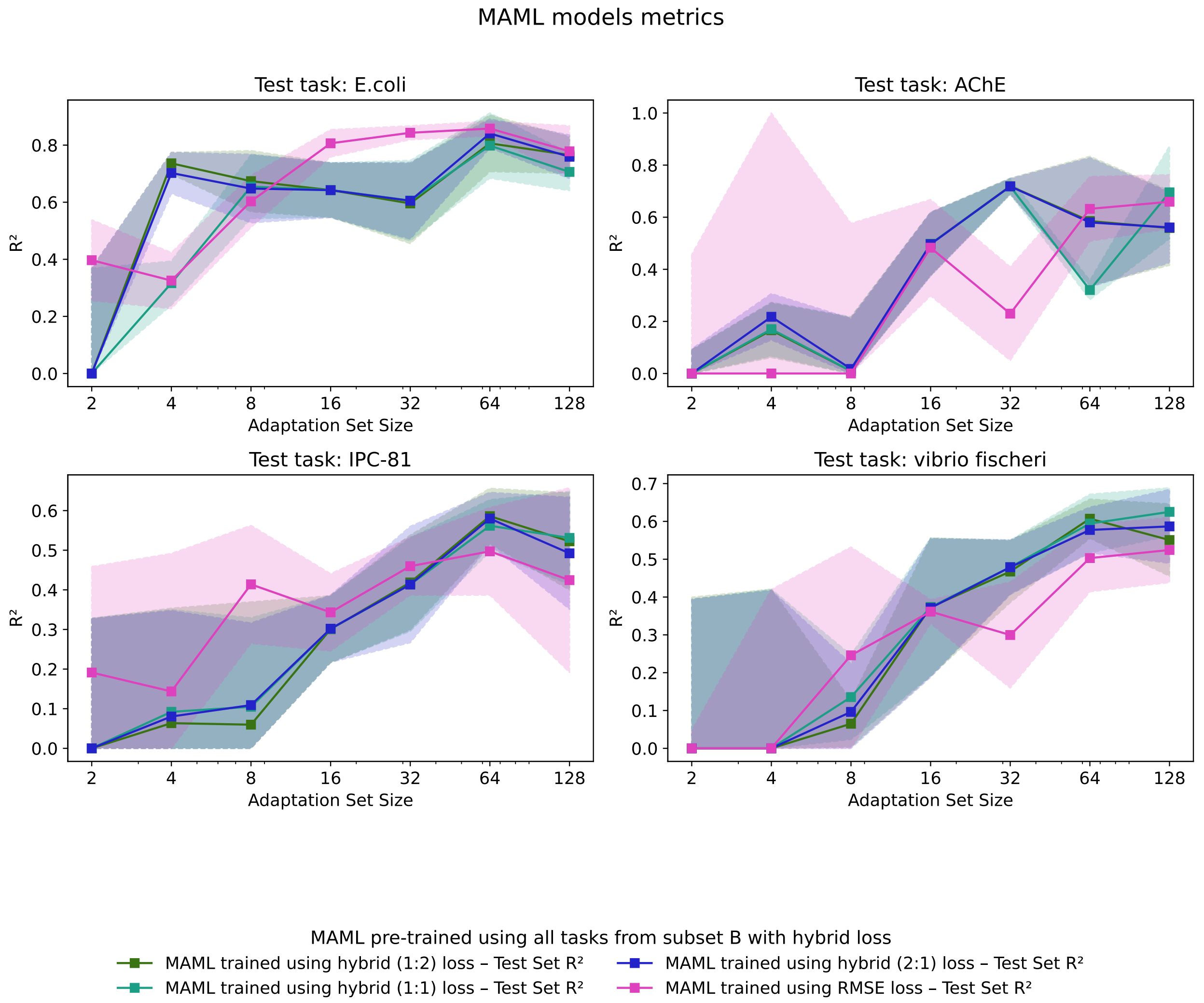

Appendix B

Metrics of MAML and Att-MAML models (without prolonged meta-training)

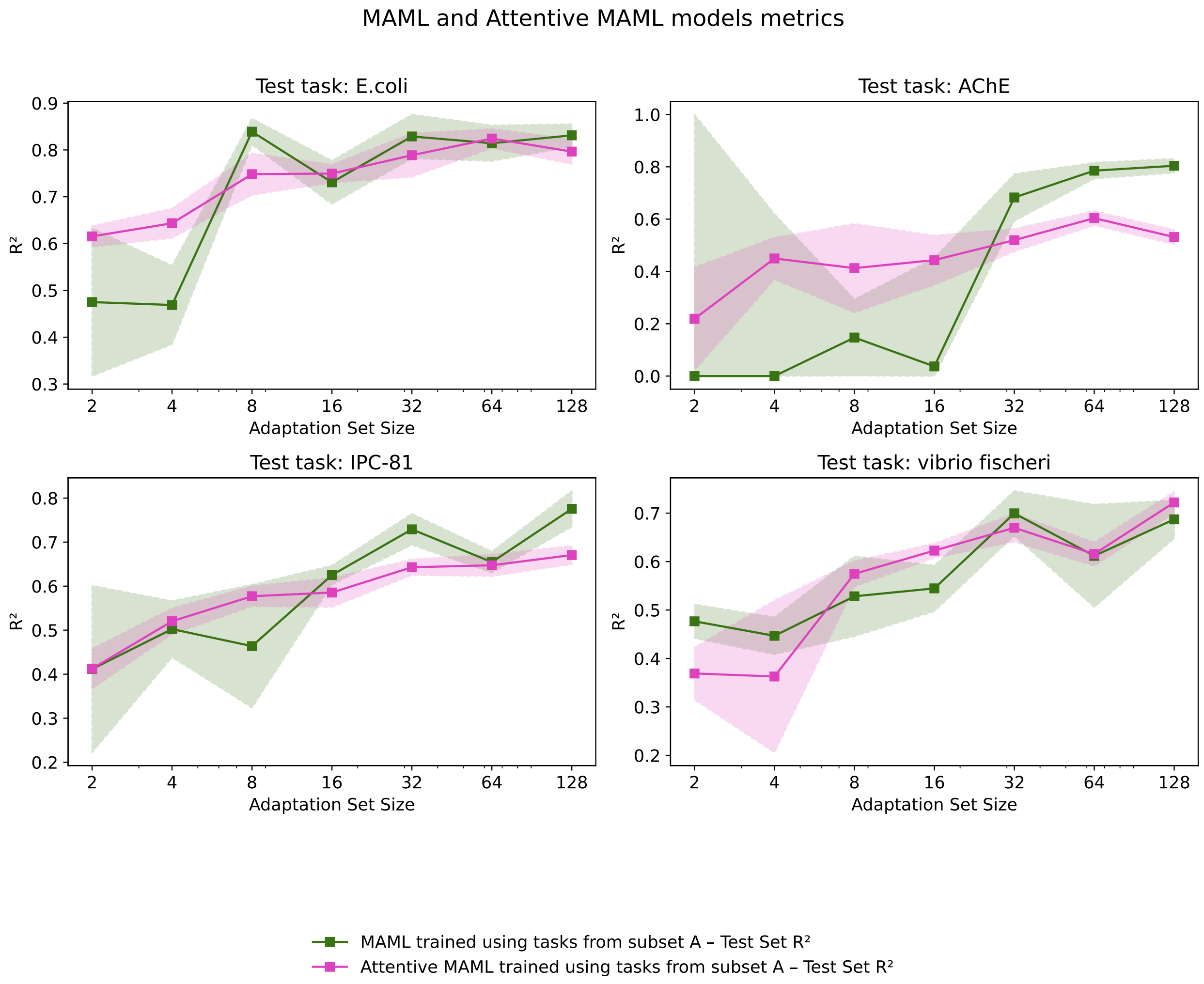

Appendix C

Metrics of MAML and Att-MAML models (with prolonged meta-training)

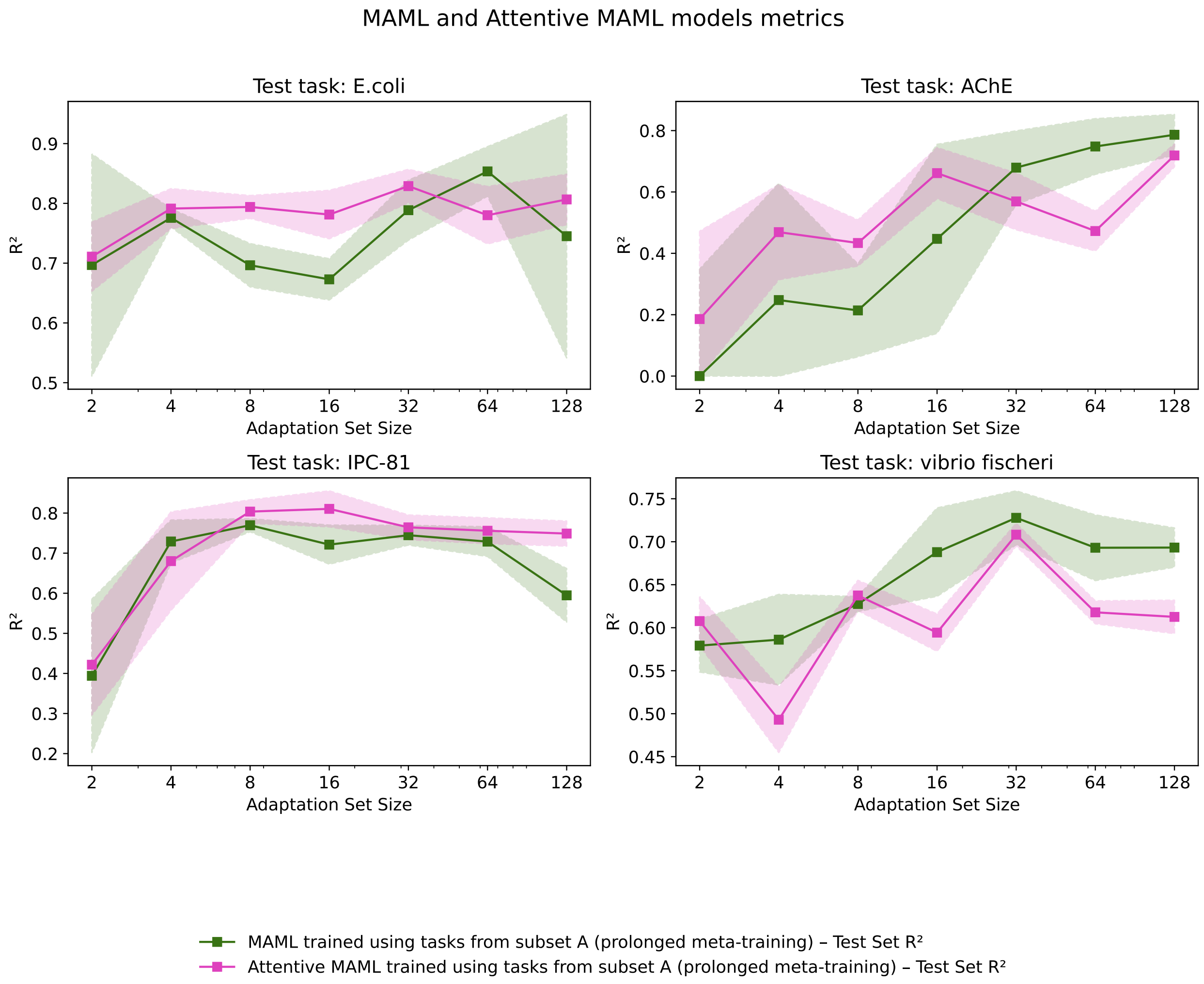

Supplement: Supplementary file 1 [file tx6c00022_si_001.pdf]
